# Spin-contrast-variation small-angle neutron scattering study of fully and partially swollen silica-filled rubber

**DOI:** 10.1107/S1600576726000361

**Published:** 2026-03-20

**Authors:** Yohei Noda, Satoshi Koizumi, Tomomi Masui, Hiroyuki Kishimoto, Daisuke Yamaguchi, Takayuki Kumada, Shin-ichi Takata, Kazuki Ohishi, Jun-ichi Suzuki

**Affiliations:** a Institute of Quantum Beam Science Ibaraki University Ibaraki 316-8511 Japan; b Neutron Industrial Application Promotion Center Comprehensive Research Organization for Science and Society (CROSS) Ibaraki 319-1106 Japan; c Research Center for Advanced Technology & Innovation, Research & Development HQ Sumitomo Rubber Industries Ltd Kobe 651-0072 Japan; d Materials Sciences Research Center Japan Atomic Energy Agency Ibaraki 319-1195 Japan; e J-PARC Center Japan Atomic Energy Agency Ibaraki 319-1195 Japan; f Neutron Science and Technology Center Comprehensive Research Organization for Science and Society (CROSS) Ibaraki 319-1106 Japan; Australian Centre for Neutron Scattering, ANSTO, Australia

**Keywords:** small-angle neutron scattering, SANS, contrast variation, dynamic nuclear polarization, silica-filled rubber, bound rubber, swelling, polymer adsorption layer

## Abstract

We have elucidated the polymer adsorption layer structure in filler–rubber systems by conducting spin-contrast-variation small-angle neutron scattering (SANS) on partially and fully swollen filler–rubber samples with and without a silane coupling agent. In spin-contrast-variation SANS, dynamic nuclear polarization (DNP) was used to polarize protons and change their scattering length with respect to polarized neutron beams significantly. SANS measurements were performed in dynamically polarized states using a DNP cryostat (1.2 K and 3.35 T). From SANS profiles obtained at various proton spin polarizations, partial scattering functions (PSFs) for each component were separated by regarding each sample as a three-component system composed of silica, polymer and deuterated toluene. To analyze the obtained PSFs in detail, we built a structure model for the silica aggregates and the surrounding polymer adsorption layer. Numerical calculation based on this model successfully reproduced the experimentally obtained PSFs, providing the structural parameters of the silica aggregates and polymer adsorption layer. The results showed a considerable difference in structural parameters between the partially and fully swollen states. For the sample with the silane coupling agent, the thickness of the polymer adsorption layer decreased as the solvent fraction increased. The difference in polymer volume fraction between the polymer adsorption layer and the outside matrix was very small in less swollen states but significant in the fully swollen state. Furthermore, the scattering contribution of the polymer chains in the solvent was accurately separated via contrast variation. In the swollen silica-filled rubber without the silane coupling agent, the size of the polymer-dense regions was almost constant, regardless of the swelling ratio. By contrast, in the swollen silica-filled rubber with the silane coupling agent, the size of the polymer-dense regions significantly increased by a factor of 2 with an increase in the swelling ratio.

## Abstract 2

We have elucidated the polymer adsorption layer structure in filler–rubber systems by conducting spin-contrast-variation small-angle neutron scattering on partially and fully swollen filler–rubber samples with and without a silane coupling agent.

## 1. Introduction

Filler–rubber systems are widely used in industrial products such as pneumatic tires, conveyer belts, shock absorbers and rubber seals. Filler particles, such as carbon and silica particles, are added to rubber to improve tear and wear resistance. A polymer adsorption layer forms around these filler particles and plays an important role in boosting mechanical performance. Due to its industrial value, the polymer adsorption layer has been investigated using various approaches, such as small-angle X-ray scattering and small-angle neutron scattering (SANS) (Baeza *et al.*, 2016 ▸; Botti *et al.*, 2006 ▸; Genix & Oberdisse, 2015 ▸; Genix *et al.*, 2019 ▸; Iwasaki *et al.*, 2025 ▸; Jimenez *et al.*, 2019 ▸; Jouault *et al.*, 2010 ▸; Liu *et al.*, 2017 ▸; Mashita *et al.*, 2013 ▸; Mashita *et al.*, 2016 ▸; Miyazaki *et al.*, 2007 ▸; Morfin *et al.*, 2006 ▸; Nakanishi *et al.*, 2021 ▸; Nakanishi *et al.*, 2024 ▸; Presto *et al.*, 2020 ▸; Shui *et al.*, 2021*a* ▸; Shui *et al.*, 2021*b* ▸; Staropoli *et al.*, 2019 ▸; Staropoli *et al.*, 2020 ▸; Takenaka *et al.*, 2009 ▸; Takenaka *et al.*, 2012 ▸; Watanabe *et al.*, 2023 ▸; Yamaguchi *et al.*, 2017 ▸), neutron reflectivity (Hori *et al.*, 2017 ▸; Kumada *et al.*, 2024 ▸; Shimokita *et al.*, 2024 ▸; Shimomura *et al.*, 2016 ▸), neutron spin echo (Jiang *et al.*, 2015 ▸; Koga *et al.*, 2018 ▸; Salatto *et al.*, 2021 ▸), mechanical analysis (Baeza *et al.*, 2014 ▸; Mujtaba *et al.*, 2014 ▸), nuclear magnetic resonance (NMR) (Chassé *et al.*, 2013 ▸; Kishimoto *et al.*, 2023 ▸; Valentín *et al.*, 2010 ▸), and atomic force microscopy (Ito *et al.*, 2022 ▸; Morozov *et al.*, 2012 ▸; Ueda *et al.*, 2019 ▸). Many researchers have combined several approaches.

Contrast-variation SANS has been successfully performed on filler–rubber systems swollen with deuterated solvents (d-solvents) (Takenaka *et al.*, 2009 ▸; Takenaka *et al.*, 2012 ▸; Liu *et al.*, 2017 ▸; Nakanishi *et al.*, 2024 ▸). Polymer confined to the filler surface absorbs less solvent than the surrounding free polymer. This solvent distribution difference can cause a contrast between the polymer adsorption layer and the surrounding free polymer. Researchers have decomposed SANS profiles at various contrasts into partial scattering functions (PSFs) to evaluate the adsorption layer thickness and the local polymer fraction in the polymer adsorption layer and the surrounding matrix. However, such studies have only been performed on systems in the fully swollen state, not those in the partially swollen state. Filler–rubber systems with different swelling ratios should be investigated for a more detailed understanding of the polymer adsorption layer. The existence of a more complex internal structure of the polymer adsorption layer is being debated. For example, Shui *et al.* (2021*a* ▸, 2021*b* ▸) and Huang *et al.* (2021 ▸) proposed a double-layer model.

**Figure 1:**
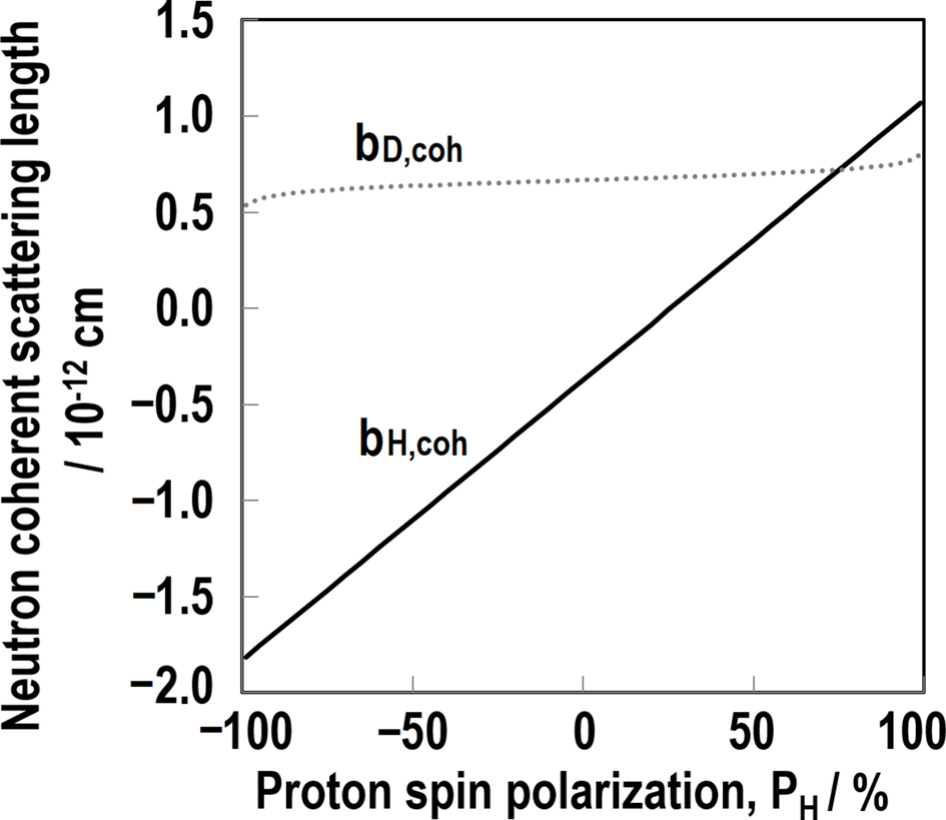
Neutron scattering lengths of protons and deuterons as a function of proton spin polarization (*P*_H_) in the case of fully polarized protons (*P*_N_ = 1).

Spin-contrast-variation SANS combined with d-solvent addition is expected to be an ideal solution for studying partially swollen filler–rubber systems. Deuterium substitution, which utilizes the neutron scattering length difference between a proton and a deuteron, is widely used. As protons and neutrons have spin, the scattering length of a proton (*b*_H_) with respect to a fully polarized neutron significantly depends on the proton spin polarization (*P*_H_) (Sears, 1992 ▸):
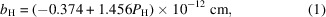
where
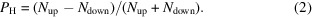
*N*_up_ and *N*_down_ mean the number of up and down proton spins, respectively. Scattering lengths can vary significantly (Fig. 1 ▸), which can be utilized for contrast variation in SANS. After the pioneering work of Stuhrmann *et al.* (1986 ▸), we successfully performed spin-contrast-variation SANS for filler–rubber systems (Noda *et al.*, 2013 ▸, Noda *et al.*, 2016 ▸).

The use of the conventional approach, namely, control of the solvent H/D ratio, to partially swollen filler–rubber systems is hindered by the following difficulties. (i) The limited amount of solvent in less swollen samples narrows the contrast-variation range. (ii) Samples with solvents with different H/D ratios must be prepared carefully, as fluctuations in the degree of swelling across samples impair the accuracy of decomposed PSFs. (iii) The incoherent scattering background tends to increase, as protonated solvents are needed to expand the contrast-variation range.

Spin-contrast-variation SANS is expected to solve these difficulties. (i) The scattering lengths of polymer chain protons can be controlled in spin-contrast-variation SANS, so contrast can be changed significantly, even for less swollen samples. Spin polarization can diffuse through flip-flops between neighboring proton spins. This process differs from the addition of d-solvents, which cannot modify the scattering length of polymer chain protons. (ii) Only one sample is necessary because a set of SANS profiles with different contrasts can be obtained by controlling *P*_H_. Fluctuations in swelling degree across samples is not a problem. (iii) Contrast can be controlled through *P*_H_, eliminating the need for a protonated solvent. This lowers the incoherent scattering background. In summary, contrast creation via d-solvent introduction and contrast variation via proton spin polarization can be combined to study partially swollen filler–rubber systems effectively.

D-solvent swelling can be used to visualize not only the polymer adsorption layer but also the polymer chain distribution. It has been used to evaluate polymer inhomogeneity and polymer network structure sizes in gel systems. Using this approach, Ikeda *et al.* (2009 ▸) studied sulfur-cross-linked rubber with different zinc oxide concentrations. Karino *et al.* (2007 ▸) and Suzuki *et al.* (2010 ▸) studied peroxide-cross-linked natural rubber containing protein aggregates, finding significant low-*q* scattering. They performed a contrast-variation study while controlling the solvent H/D ratio and isolated the scattering contribution of the solvent polymer chains from the contribution of protein aggregates. For swollen silica-filled rubber samples, the target material of the current study, the high-*q* scattering of the polymer network structure (mesh size) can be evaluated easily because the scattering contribution of silica particles is small at high *q*. However, the low-*q* scattering contribution of polymer distribution inhomogeneity is difficult to evaluate because of its overlap with the silica particle contribution. In the present study, we attempt to use contrast variation to separate the scattering contribution of polymer chains in swollen silica-filled rubber samples. Specifically, we applied spin-contrast-variation SANS to partially and fully swollen silica-filled rubber samples, focusing on the effect of a silane coupling agent.

## 2. Experimental

### 2.1. Sample preparation

**Figure 2:**
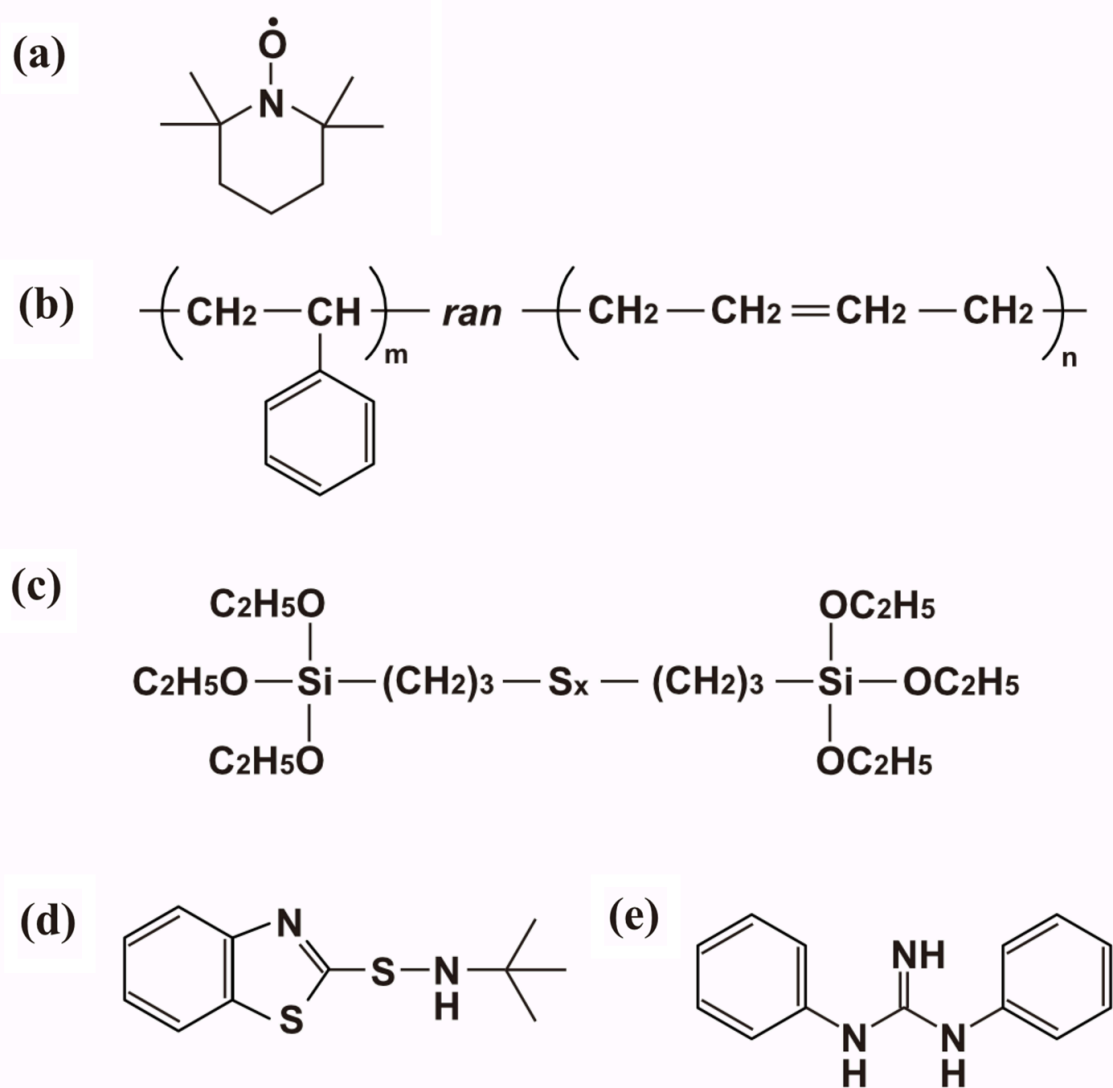
Molecular structure formulas of TEMPO (*a*), SBR (*b*), silane coupling agent Si266 (*c*), TBBS (*d*) and DPG (*e*). In (*c*), the average sulfur chain length (*x*) is 2.15.

We studied the polymer adsorption layer around silica particles by preparing two samples without and with a silane coupling agent [Si266; Fig. 2 ▸(*c*)], labeled R_N_ and R_CA_, respectively. Table 1 ▸ shows the sample composition. Both R_N_ and R_CA_ contain 5 vol.% silica particles in a styrene–butadiene random copolymer [SBR; Fig. 2 ▸(*b*)]. In addition, sulfur, *N*-*tert*-butyl-2-benzo­thia­zole sulfenamide [TBBS; Fig. 2 ▸(*d*)] and 1,3-di­phenyl­guanidine [DPG; Fig. 2 ▸(*e*)] were added for vulcanization. All ingredients were mixed in a milling machine, and the resulting mixture was pressed into molds and heated at 170°C for 12 min. The thickness of the rubber sheet before swelling was approximately 0.6 mm for R_N_ and about 0.9 mm for R_CA_.

Samples in dynamic nuclear polarization (DNP) experiments should include unpaired electrons. The stable free radical 2,2,6,6-tetra­methyl­piperidin-1-oxyl [TEMPO; Fig. 2 ▸(*a*)] can be used conveniently as an electron spin source (Bunyatova, 2004 ▸). In our previous study on filler–rubber systems without solvents, we used vapor sorption, spontaneously diffusing TEMPO vapor into rubber (Noda *et al.*, 2016 ▸). TEMPO doping was easily completed by adding TEMPO to the solvent for swelling. The optimal TEMPO concentration is 30 m*M* for DNP experiments at 3.35 T (Noda *et al.*, 2016 ▸), which is much lower than the solvent amount needed for swelling. In addition, the O_2_ concentration in a sample should be minimized to obtain a high |*P*_H_| in DNP experiments. O_2_ accelerates proton spin relaxation. Considering these issues, we devised the current experimental procedure as follows.

For the swelling of rubber samples, we used deuterated toluene (d-toluene, 99 at.% D; Sigma–Aldrich). To prepare the partially swollen samples, we placed a cut rubber sheet (10.5 × 10.5 mm) of R_N_ or R_CA_ in an aluminium metallized film package. Then, a TEMPO/d-toluene solution (73–109 m*M*) was added into the package using a microsyringe. By adjusting the solution volume (0.03–0.10 ml), we obtained swollen samples with *Q*_swell_ ≃ 1.5 for R_N1_ and R_CA1_ and *Q*_swell_ ≃ 2 for R_N2_ and R_CA2_, where *Q*_swell_ is the ratio of the swollen rubber volume to the initial one. Then, we placed an oxygen absorber (A500-HS, AS ONE) in the package and sealed it using a heat sealer. The package containing the swollen sample was kept at room temperature for 12 h before the SANS experiment. The *Q*_swell_ values in the saturated state were 5.51 and 4.76 for R_N_ and R_CA_, respectively.

To prepare the fully swollen samples (R_N3_ and R_CA3_), we placed a cut rubber sheet (7 × 7 mm) of R_N_ or R_CA_ in a glass bottle with a sealing cap. Then, an excess amount (approximately 0.25 ml) of a TEMPO/d-toluene solution (48 m*M*), compared with the cut rubber sheet, was added to the bottle. We placed an oxygen absorber in the package while avoiding its contact with the solution. The glass bottle containing the fully swollen sample was kept at room temperature for 12 h before the SANS experiment.

According to electron spin resonance measurements, the TEMPO concentrations of R_N1_, R_N2_, R_CA1_ and R_CA2_ were 34, 28, 31 and 37 m*M*, respectively. They were close enough to the optimal TEMPO concentration (30 m*M*) for DNP experiments at 3.35 T. Loss of TEMPO during swelling was insignificant (approximately 15% at most). Given the confirmed reliability of this procedure, the TEMPO concentration evaluation of R_N3_ and R_CA3_ was omitted. For SANS, we prepared samples in different packages in a similar way.

A sample obtained from each package was immediately placed in a sample-holding unit at the end of the sample stick of a DNP cryostat (Section 2.2). Then, the unit was inserted into the sample chamber of the DNP cryostat, which was filled with liquid He.

### 2.2. Dynamic nuclear polarization

At thermal equilibrium, proton spins are polarized slightly (*P*_H_ = 0.30%), even at 1.2 K and 3.35 T. However, under the same conditions, electron spins are almost fully polarized (95%). In DNP, microwave irradiation stimulates polarization transfer from electron to proton spins to achieve a high *P*_H_ (Abragam & Goldman, 1978 ▸). Here, we used a DNP cryostat (Kumada *et al.*, 2009 ▸) designed for SANS, which had split-type superconducting magnet coils. Between the coils is a sample chamber, which is filled with liquid ^4^He, and its temperature can be reduced to 1.2 K by evacuating liquid ^4^He. The magnetic field is parallel to the direction of the neutron beam, which passes along the central axis of the magnet coils. Thin aluminium plates form windows through which the neutron beam passes, causing a slight SANS background.

Each sample was placed in the sample-holding unit at the end of the sample stick of the DNP cryostat. The sample-holding unit had a three-turn NMR coil for *P*_H_ evaluation. The sample sheet (14 × 14 × ∼1 mm) was inserted in this NMR coil. The NMR signal was proportional to *P*_H_, so we could simultaneously measure NMR during the SANS experiment. The NMR coil was made of a 0.1 mm-thick aluminium sheet and caused a slight SANS background. When protons are polarized via DNP, their NMR signal becomes significant. Then, *P*_H_ can be evaluated accurately in relative terms. To obtain absolute *P*_H_ values, we needed to calibrate *P*_H_ using thermal equilibrium NMR signals (*P*_H_ = 0.082% at 4.2 K and 3.35 T, for example). However, measurement of this NMR signal was not completed because of the low sensitivity of our NMR equipment and limited SANS beam time. Nonetheless, neutron transmission depends on *P*_H_. Therefore, we calibrated *P*_H_ through neutron transmission (Section 2.3).

Microwave radiation for polarization transfer from electron to proton spins was generated using a Gunn oscillator (94 GHz) placed on the top plate of the DNP cryostat, which irradiated the sample through a stainless-steel pipe with a length of 1 m and an inside diameter of 6 mm. *P*_H_ was controlled through microwave frequency tuning, as *P*_H_ quickly responds to changes in microwave conditions. The time constant for this response was approximately 3 min.

### 2.3. Small-angle neutron scattering

SANS experiments were performed using TAIKAN (BL15) (Shinohara *et al.*, 2009 ▸; Shinohara *et al.*, 2009 ▸; Takata *et al.*, 2015 ▸) at the Material and Life Science Experimental Facility (MLF) in the Japan Proton Accelerator Research Complex (J-PARC). The DNP cryostat was placed on the TAIKAN sample stage. The device allocation is described in our previous article (Noda *et al.*, 2016 ▸). Because of the DNP cryostat window structure, the available scattering angle 2θ was limited (2θ < 15°) and the detectors could not be fully utilized. A polarized neutron beam was provided by a magnetic supermirror polarizer composed of an Fe/Si multilayer. Since the neutron polarization decreases for short-wavelength neutrons (λ < 4 Å), we employed SANS data with a limited wavelength range (4 < λ < 7.6 Å), where the magnitude of neutron polarization was close enough to 1, resulting in a *q* range of 0.005 < *q* < 0.3 Å^−1^ as in our previous study (Noda *et al.*, 2016 ▸). SANS at *P*_H_ = 0% does not depend on neutron polarization. We will employ the SANS data at *P*_H_ = 0% for the full wavelength range of neutrons (1 < λ < 7.6 Å) to discuss the high-*q* profile (*q* < 1 Å^−1^) later in Section 3.5.

As TAIKAN simultaneously measures SANS and neutron transmission, we used the observed neutron transmission to calibrate *P*_H_. The microscopic total cross sections for H, D, C, N, O, S and Si are σ_tot,H_ = (81.99 − 66.97*P*_H_) × 10^−24^ cm^2^, σ_tot,D_ = (7.63 + 3.76*P*_D_) × 10^−24^ cm^2^, σ_tot,C_ = 5.55 × 10^−24^ cm^2^, σ_tot,N_ = 13.41 × 10^−24^ cm^2^, σ_tot,O_ = 4.23 × 10^−24^ cm^2^, σ_tot,S_ = 1.56 × 10^−24^ cm^2^ and σ_tot,Si_ = 2.34 × 10^−24^ cm^2^, respectively (Sears, 1992 ▸). Here, *P*_D_ is deuteron spin polarization. The macroscopic total cross section (Σ_tot_) of each ingredient and each sample was calculated using the known chemical compositions (Tables 2 ▸ and 3 ▸).

**Figure 3:**
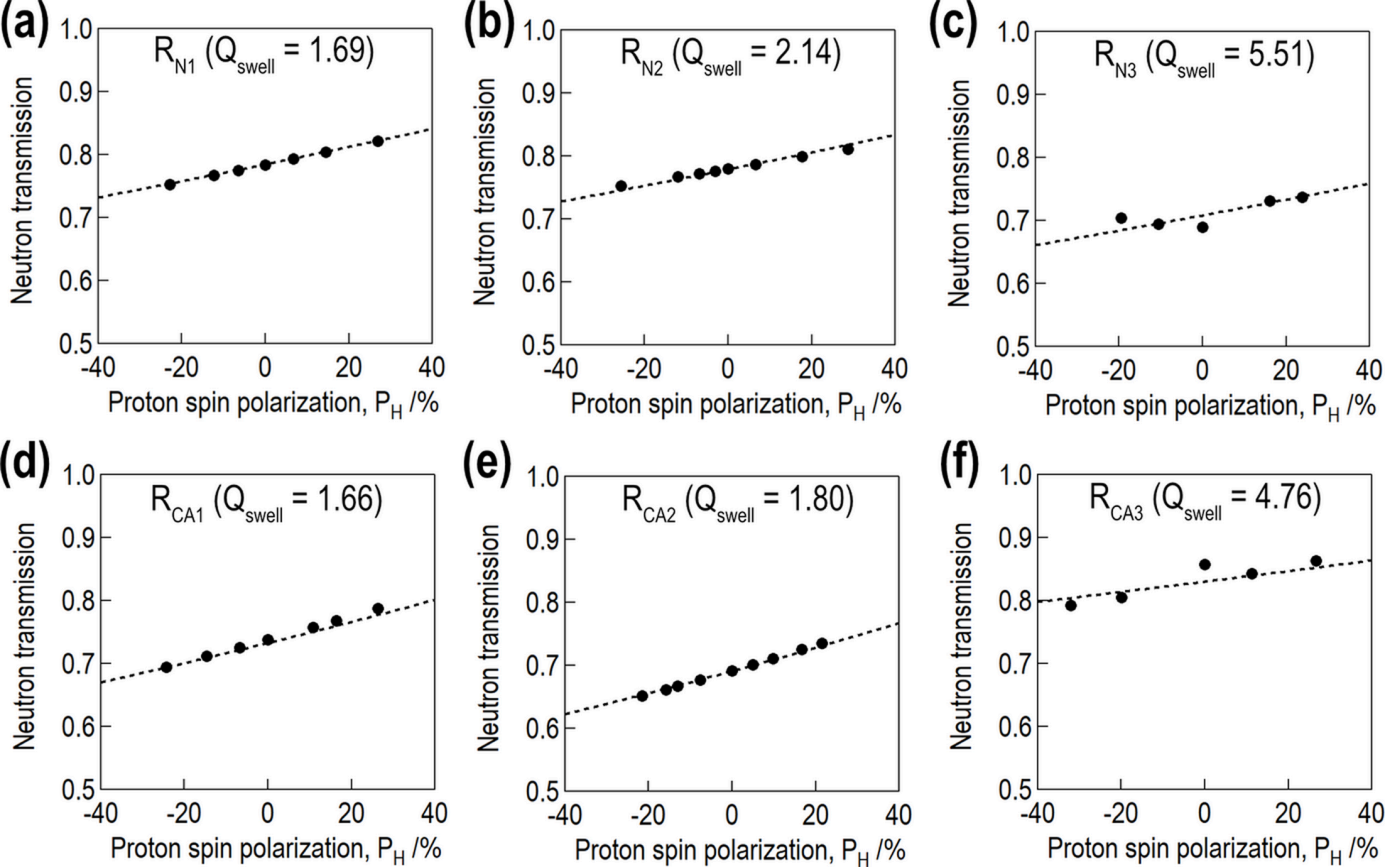
Neutron transmission as a function of *P*_H_. The top and bottom rows present the results for the rubber samples without and with the silane coupling agent, respectively. In each panel, the filled black circles indicate the experimental results, and the dased lines indicate the numerical calculation results.

Protons and deuterons have common spin temperatures, even in dynamically polarized states (de Boer *et al.*, 1974 ▸). In this case, *P*_D_ is related to *P*_H_ by
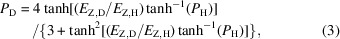
where *E*_Z,D_ and E_Z,H_ are the Zeeman splitting energies of protons and deuterons, respectively, and *E*_Z,D_/*E*_Z,H_ is 0.1535. Under our experimental conditions (|*P*_H_| < 30%), tanh^−1^(*P*_H_) could be approximately regarded as *P*_H_. Hence, we used the approximate equation
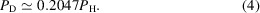
Neutron transmission *T*_N_ was calculated as
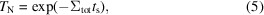
where *t*_s_ is sample thickness. We calibrated *P*_H_ by comparing the calculated and experimental *T*_N_. In Fig. 3 ▸, the filled black circles indicate the experimentally obtained *T*_N_ as a function of *P*_H_ after calibration, where *T*_N_ increased with *P*_H_. The less swollen samples had steeper slopes than the fully swollen samples, as the latter had a higher proton concentration.

## 3. Results and discussion

### 3.1. Small-angle neutron scattering results

**Figure 4:**
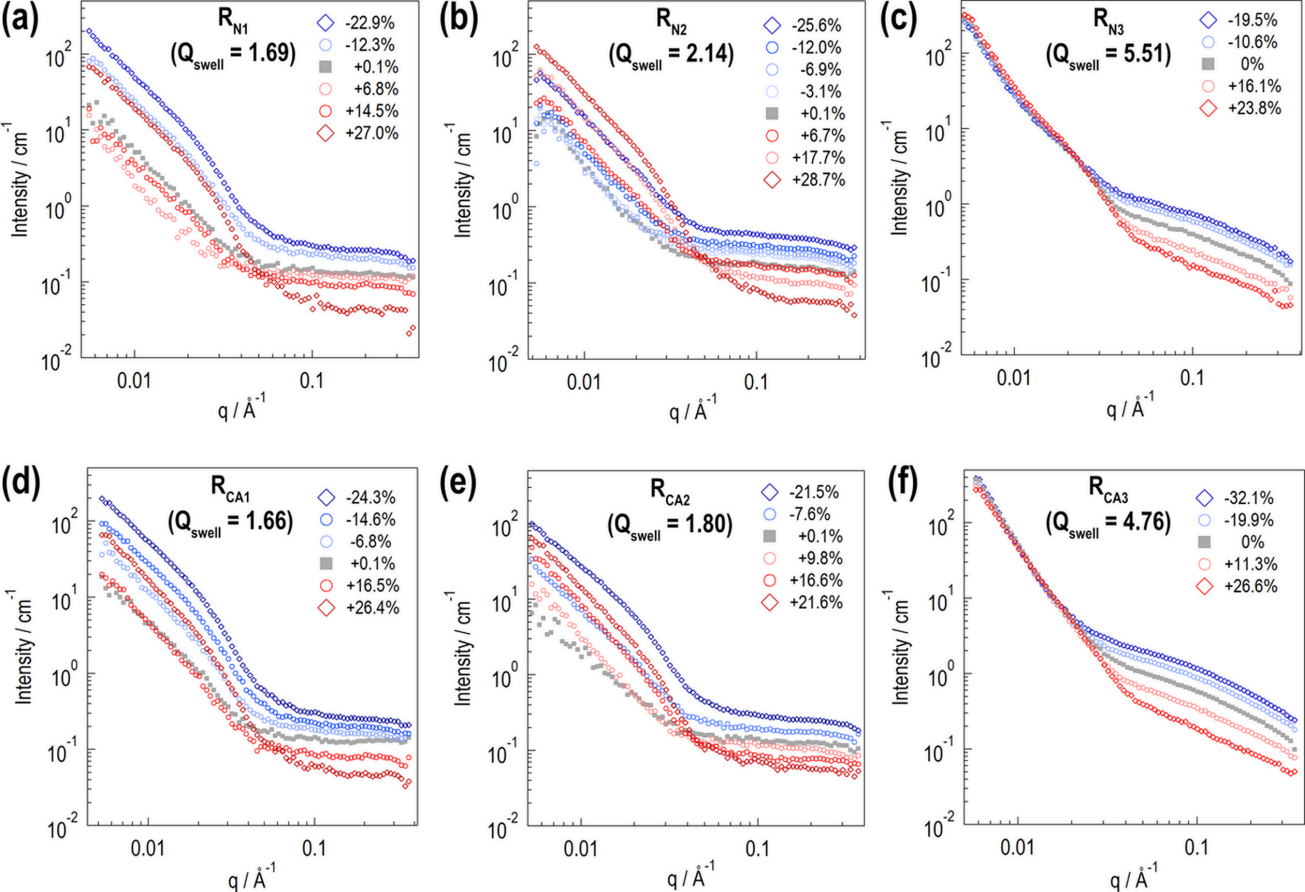
SANS profiles observed at various *P*_H_. The horizontal axis shows the magnitude of the scattering vector *q* [= (4π/λ) sin θ, where λ is the neutron wavelength and 2θ is the scattering angle]. The top and bottom rows present the results for the rubber samples without and with the silane coupling agent, respectively. In each profile, the incoherent scattering intensity contribution is subtracted.

Fig. 4 ▸ shows SANS profiles observed at various *P*_H_ for the silica-filled swollen rubber samples. The horizontal axis is the magnitude of the scattering vector *q* [= (4π/λ) sin θ]. In each panel, the filled gray symbols indicate SANS profiles before DNP. After DNP, the proton spins are polarized by up to several tens in percentage. The red and blue symbols indicate SANS profiles in positively and negatively polarized states, respectively. A significant profile change is thus successfully demonstrated.

The profiles in Fig. 4 ▸ exclude incoherent scattering contributions. These incoherent scattering contributions are evaluated as follows. The microscopic incoherent scattering cross sections for H and D are σ_inc,H_ = 26.64(3 − 2
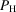
 − 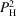) × 10^−24^ cm^2^ and σ_inc,D_ = 1.02(2 − 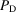 − 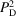) × 10^−24^ cm^2^, respectively (Sears, 1992 ▸). Contributions from other atoms are negligible. As *P*_H_ increases, incoherent scattering decreases monotonically. The macroscopic incoherent scattering cross section (Σ_inc_) of each ingredient and each sample is calculated using the known chemical compositions (Tables 2 ▸ and 3 ▸). From the observed SANS profiles, we subtract the incoherent scattering intensity *I*_inc_, which is calculated considering multiple scattering (Shibayama *et al.*, 2005 ▸) as
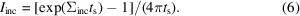


For the less swollen samples [Figs. 4 ▸(*a*), 4 ▸(*b*), 4 ▸(*d*) and 4 ▸(*e*)], a prominent scattering contribution is found at low *q* (*q* < 0.03 Å^−1^), which is probably due to silica aggregates. The low-*q* contribution exhibits quadratic *P*_H_ dependence, with its minimum being at around *P*_H_ = 0%. A less steep contribution is found at high *q* (*q* > 0.03 Å^−1^), which is due to the polymer chains in the d-solvent. The high-*q* contribution decreases monotonically as *P*_H_ increases. For the fully swollen samples [Figs. 4 ▸(*c*) and 4 ▸(*f*)], the low-*q* contribution does not change significantly, whereas the high-*q* contribution decreases monotonically as *P*_H_ increases.

The scattering intensity is proportional to the contrast factor, which is the squared difference in scattering length density (SLD) between relevant domains. The coherent scattering lengths for H, D, C, N, O, S and Si are *b*_H_ = (−0.374 + 1.456*P*_H_) × 10^−12^ cm, *b*_D_ = (0.667 + 0.27*P*_D_) × 10^−12^ cm, *b*_C_ = 0.665 × 10^−12^ cm, *b*_N_ = 0.936 × 10^−12^ cm, *b*_O_ = 0.580 × 10^−12^ cm, *b*_S_ = 0.285 × 10^−12^ cm and *b*_Si_ = 0.415 × 10^−12^ cm, respectively (Sears, 1992 ▸). The SLD of each ingredient in the swollen filler–rubber samples is calculated using their known chemical compositions (Table 2 ▸). The swollen silica-filled rubber is a three-component system composed of silica, polymer and d-toluene. The SLD of each component (ρ_S_ for silica, ρ_P_ for polymer, ρ_T_ for d-toluene) is
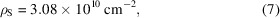

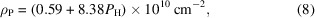

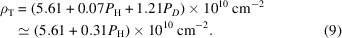


**Figure 5:**
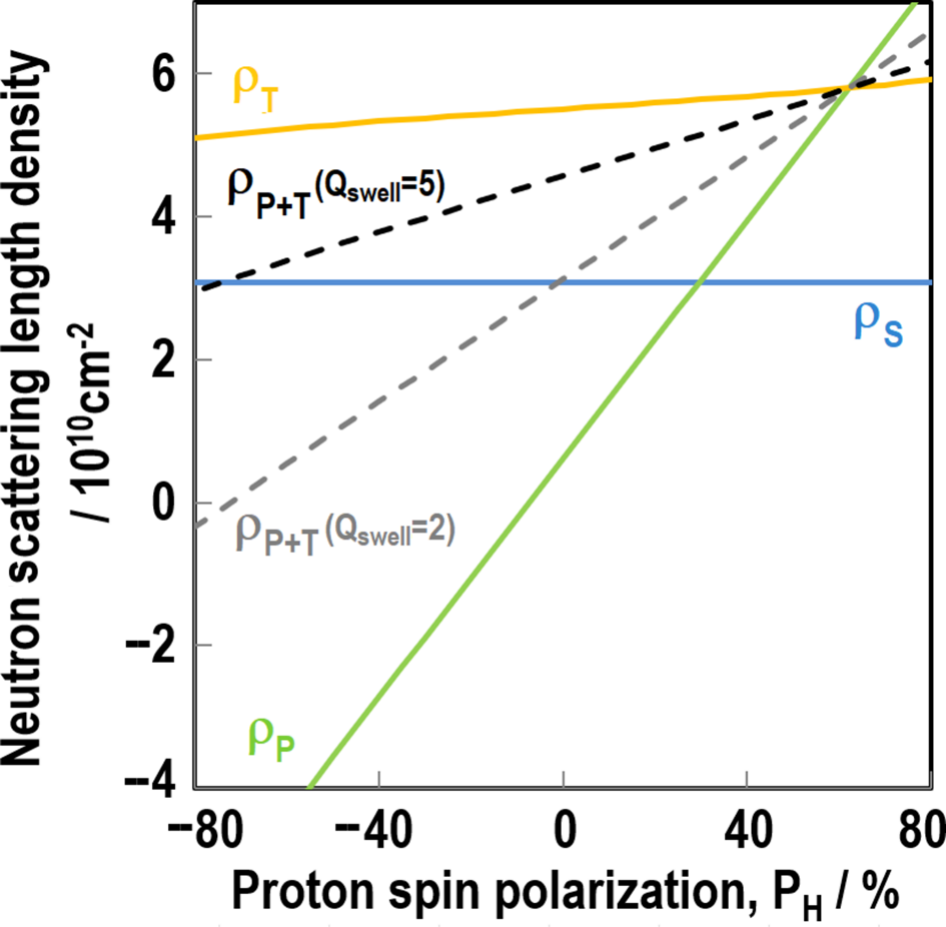
Neutron SLDs of three components as a function of *P*_H_. The blue, green and orange solid lines represent neutron SLDs of silica (ρ_S_), polymer (ρ_P_) and d-toluene (ρ_T_), respectively. The gray and black dashed lines represent neutron SLDs calculated for a homogeneous mixture of polymer and d-toluene (ρ_P+T_) at *Q*_swell_ = 2 and *Q*_swell_ = 5, respectively.

To calculate ρ_P_, we considered only the contributions of the SBR and sulfur. We ignored the contributions of the minor components (the silane coupling agent, accelerator and TEMPO), whose *P*_H_ dependence is close to that of the SBR. Fig. 5 ▸ shows the calculated SLDs as a function of *P*_H_. Before swelling, ρ_S_ and ρ_P_ match at *P*_H_ = 30%.

In Fig. 5 ▸, the gray and black dashed lines indicate SLDs for a homogeneous mixture of polymer and d-toluene (ρ_P+T_) at *Q*_swell_ of 2 and 5, respectively. At *Q*_swell_ = 2, the calculated ρ_P+T_ and ρ_S_ match at around *P*_H_ = 0%. This explains the observed results for the less swollen samples. The minimum intensity of the low-*q* contribution is at around *P*_H_ = 0%. At *Q*_swell_ = 5, the calculated ρ_P+T_ and ρ_S_ match at around *P*_H_ = −75%, which exceeds our experimental *P*_H_ range. This explains the observed results for the fully swollen samples, in which the low-*q* contribution does not change significantly with *P*_H_.

The scattering contribution of the polymer chains in d-toluene is proportional to the contrast factor (ρ_P_ − ρ_T_)^2^. ρ_P_ and ρ_T_ match at approximately *P*_H_ = 62%, which is out of our experimental *P*_H_ range. This explains the observed high-*q* scattering contribution, which monotonically decreases as *P*_H_ increases.

### 3.2. Partial scattering function

**Figure 6:**
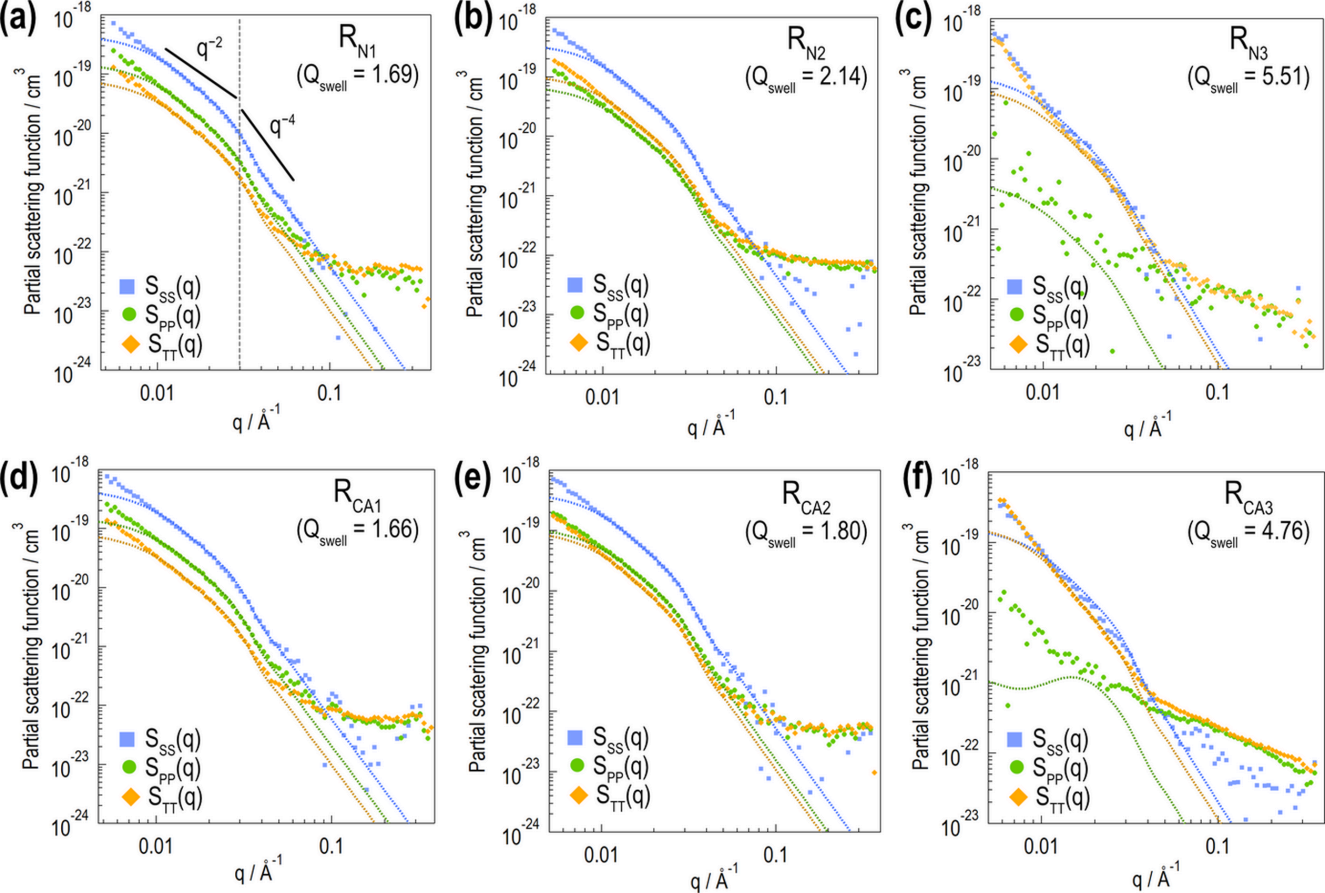
The obtained partial scattering functions. The top and bottom rows present the results for the rubber samples without and with the silane coupling agent, respectively. In each panel, the blue squares, green circles and orange diamonds indicate the experimentally obtained *S*_SS_(*q*), *S*_PP_(*q*) and *S*_TT_(*q*), respectively. The *S*_SS_(*q*), *S*_PP_(*q*) − *S*_G_(*q*) and *S*_TT_(*q*) − *S*_G_(*q*) profiles numerically calculated from the sphere collection model are shown by the dotted-line curves.

The swollen silica-filled rubber is a three-component system composed of silica, polymer and d-toluene. The spatial distribution function of the SLD is
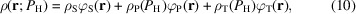
where φ_S_(**r**), φ_P_(**r**) and φ_T_(**r**) are the spatial distribution functions of silica, polymer and d-toluene, respectively. Then, the scattering intensity, *I*(*q*), is calculated as
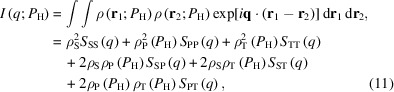
where *S_ij_*(*q*) is the PSF between components *i* and *j*,
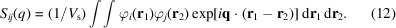
*V*_s_ is sample volume. From the above definition, *S_ij_*(*q*) = *S_ji_*(*q*). The observed SANS profiles are decomposed into PSFs following Endo (2006 ▸) and Endo *et al.* (2008 ▸) as follows. Through the incompressibility theorem, the following equation should be satisfied:
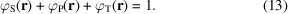


Given the definition of PSFs, the following equations are obtained:
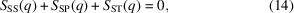

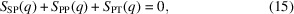

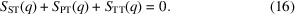
Eliminating the PSFs related to the d-toluene component results in the following equation:
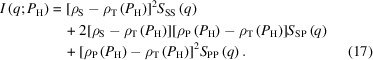


SANS profiles with various *P*_H_ are obtained via contrast-variation experiments. The PSFs are automatically obtained through the procedure described in Appendix *A*[App appa]. Consequently, *S*_SS_(*q*), *S*_PP_(*q*) and *S*_TT_(*q*) [= *S*_SS_(*q*) + *S*_PP_(*q*) + 2*S*_SP_(*q*)] are obtained (Fig. 6 ▸). *S*_SS_(*q*) exhibits a moderate slope (∼*q*^−2^) at low *q* (*q* < 0.03 Å^−1^), whereas a steep slope (∼*q*^−4^) is identified at high *q* (*q* > 0.03 Å^−1^). The low-*q* slope (∼*q*^−2^) indicates aggregate formation by silica primary particles. The obtained *S*_SS_(*q*) profiles are similar for all samples.

For the less swollen samples (R_N1_, R_N2_, R_CA1_ and R_CA2_), *S*_PP_(*q*) and *S*_TT_(*q*) exhibit similar *q* dependence with *S*_SS_(*q*) at low *q*. This is attributed to the Babinet principle as follows. We assume that the polymer and d-toluene volume fractions are φ_homo_ and (1 − φ_homo_), respectively, and that they mix homogeneously. Then, we obtain φ_P_(**r**) = φ_homo_[1 − φ_S_(**r**)] and φ_T_(**r**) = (1 − φ_homo_)[1−φ_S_(**r**)]. Hence, *S*_PP_(*q*) = 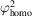
*S*_SS_(*q*) and *S*_TT_(*q*) = (1 − φ_homo_)^2^*S*_SS_(*q*).

According to the profiles of R_N2_, R_CA1_ and R_CA2_, the low-*q* slope of *S*_TT_(*q*) is slightly steeper than that of *S*_SS_(*q*), whereas that of *S*_PP_(*q*) is slightly less steep than that of *S*_SS_(*q*). This indicates deviation from the assumed homogeneous distribution of the polymer and d-toluene in the matrix and is attributed to the formation of a polymer adsorption layer (Section 3.3).

Unlike *S*_SS_(*q*), *S*_PP_(*q*) and *S*_TT_(*q*) indicate similar significant scattering contributions at high *q*. At the length scale of this high-*q* region, the spatial distribution of the polymer and d-toluene components is in an inverse relationship [φ_P_(**r**) = 1 − φ_T_(**r**)]. Hence, the Babinet principle explains the similarity between *S*_PP_(*q*) and *S*_TT_(*q*) at high *q*.

For the fully swollen samples (R_N3_ and R_CA3_), the low-*q* profile of *S*_TT_(*q*) increases, approaching that of *S*_SS_(*q*). By contrast, *S*_PP_(*q*) decreases. This is reasonably understood through the increase in the d-toluene volume fraction and the decrease in the polymer volume fraction outside the silica. The *S*_SS_(*q*) and *S*_TT_(*q*) profiles of R_N3_ are similar at low *q*, whereas *S*_SS_(*q*) and *S*_TT_(*q*) of R_CA3_ differ considerably at low *q*. This is attributed to the formation of a polymer adsorption layer (Section 3.3).

Compared with the less swollen samples, the fully swollen ones show greater PSF fluctuations. This can be understood by considering the *P*_H_ dependence of the SLD. In the less swollen state (*Q*_swell_ = 2), ρ_S_ and ρ_P+T_ match at around *P*_H_ = 0 (Fig. 5 ▸). Hence, the scattering intensity significantly changes through its minimum. In the fully swollen state (*Q*_swell_ = 5), ρ_S_ and ρ_P+T_ match at approximately *P*_H_ = −70%, which is beyond our achievable range (|*P*_H_| < 30%). Therefore, the scattering intensity changes insignificantly in relative terms. As for the fully swollen state, additional SANS measurements near the matching point should provide PSFs with fewer fluctuations. For this purpose, SANS measurement at high |*P*_H_| values is beneficial. This can be achieved using a recently developed high-*P*_H_-performance DNP instrument (1.2 K, 6.7 T) designed for BL20 iMATERIA (Noda *et al.*, 2020 ▸).

### 3.3. Partial scattering function ratio

The previous section discusses the decomposed PSFs, focusing on low-*q* behavior. For R_N1_, *S*_PP_(*q*), *S*_TT_(*q*) and *S*_SS_(*q*) exhibit common *q* dependence at low *q*. Therefore, the polymer and d-toluene are homogeneously mixed in the matrix. The different *q* dependence of *S*_SS_(*q*), *S*_PP_(*q*) and *S*_TT_(*q*) of R_N2_, R_CA1_, R_CA2_ and R_CA3_ at low *q* suggests the formation of a polymer adsorption layer. This section focuses on the ratio between PSFs as a useful indicator.

**Figure 7:**
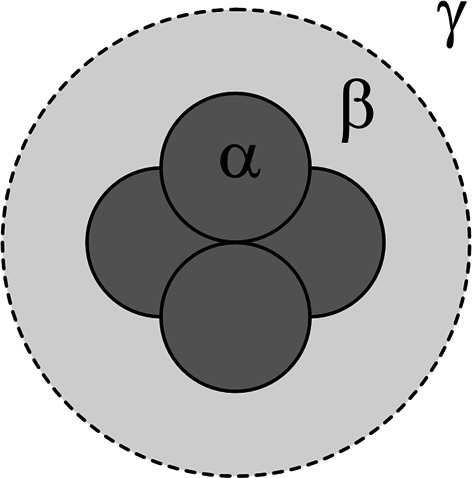
Schematic diagram of a silica aggregate surrounded by a polymer adsorption layer. The dark gray region (α) is the silica aggregate, the light gray region (β) is the polymer-dense layer, and the white external region (γ) is the matrix.

The microscopic view of swollen silica-filled rubber in Fig. 7 ▸ can be divided into three regions: the silica aggregate (α), polymer adsorption layer (β) and matrix (γ). For these three regions, we define spatial distribution functions φ_α_(**r**), φ_β_(**r**) and φ_γ_(**r**), whose value is 1 within the corresponding region and 0 otherwise. If the polymer volume fraction is φ_L_ in region β and φ_M_ in region γ, the spatial distribution functions φ_S_(**r**), φ_P_(**r**) and φ_T_(**r**) are
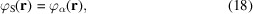

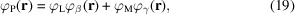

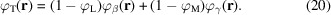
Hence, for the present three-component system, the PSFs are calculated as
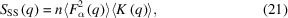

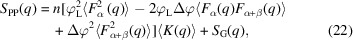

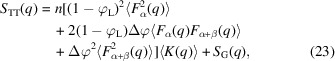

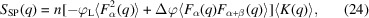

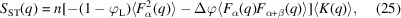

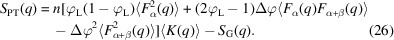
Here Δφ is the difference in polymer volume fraction (= φ_L_ − φ_M_). *n* is the number density of the aggregate. 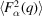 is the form factor of region α. 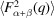 is the form factor of region α+β. 〈*F*_α_(*q*)*F*_α+β_(*q*)〉 is a cross term between regions α and α+β. The angle brackets indicate an ensemble average, accounting for polydispersity in structural parameters. 〈*K*(*q*)〉 is a structure factor accounting for the spatial distribution of the aggregate (Appendix *B*[App appb]). *S*_G_(*q*) is the scattering intensity due to the polymer chains in the solvent. The form factors and the cross term are defined as
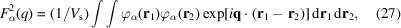

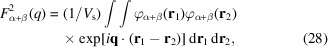

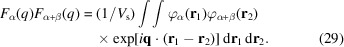
Here, *V*_s_ is the sample volume. φ_α+β_(**r**) is the spatial distribution function of region α+β [= φ_α_(**r**) + φ_β_(**r**)]. *S*_G_(*q*) is the sum of the Debye–Bueche (DB) and Ornstein–Zernike (OZ) functions (Debye & Bueche, 1949 ▸; Ornstein & Zernike, 1914 ▸),
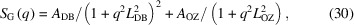
where *L*_DB_ is the size of cross-linking density heterogeneity and *L*_OZ_ is the mesh size of the polymer network. For a swollen rubber sample without filler particles, the corresponding PSFs 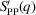, 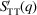 and 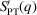 are
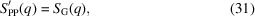

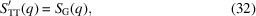

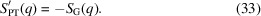
These contributions are simply incorporated into *S*_PP_(*q*), *S*_TT_(*q*) and *S*_PT_(*q*) as in previous studies (*e.g.* Nakanishi *et al.*, 2024 ▸).

We propose the ratio −*S*_SP_(*q*)/*S*_SS_(*q*) as a useful indicator of the formation of the polymer adsorption layer:
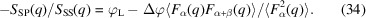


Our use of this ratio avoids the effect of 〈*K*(*q*)〉 and *S*_G_(*q*), enabling least-squares fitting in a wider *q* range with fewer adjustable parameters. In equation (34), the second term on the right side, 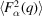, is an ordinary form factor that decreases according to Porod’s law (*q*^−4^) at high *q*. 〈*F*_α_(*q*)*F*_α+β_(*q*)〉 is cross term that is a product of oscillation functions with different frequencies. As *q* increases, the cross term quickly reduces to a negative value. Hence, 〈*F*_α_(*q*)*F*_α+β_(*q*)〉 drops faster than 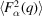 at high *q*. The contribution of the second term diminishes to provide a constant φ_L_.

The meaning of equation (34) is clarified using the following approximate equations based on the Guinier approximation, which are satisfied only at low *q*:
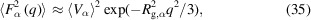

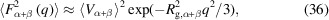

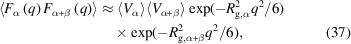
where 〈*V*_α_〉 and 〈*V*_α+β_〉 are the expected volumes of regions α and α+β, respectively. *R*_g,α_ and *R*_g,α+β_ are the gyration radii of regions α and α+β, respectively. Applying these to equation (34) yields the approximate equation
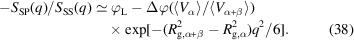


−*S*_SP_(*q*)/*S*_SS_(*q*) is expected to show a flat region (= φ_L_) at high *q* and a depression at low *q*. This approximate equation provides an opportunity to evaluate Δφ(〈*V*_α_〉/〈*V*_α+β_〉) as the depression depth and (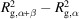) by the *q* dependence of the depression curve. −*S*_ST_(*q*)/*S*_SS_(*q*) can be used for the same purpose. However, the sum of −*S*_SP_(*q*)/*S*_SS_(*q*) and −*S*_ST_(*q*)/*S*_SS_(*q*) is 1. Hence, only one of them should be assessed.

**Figure 8:**
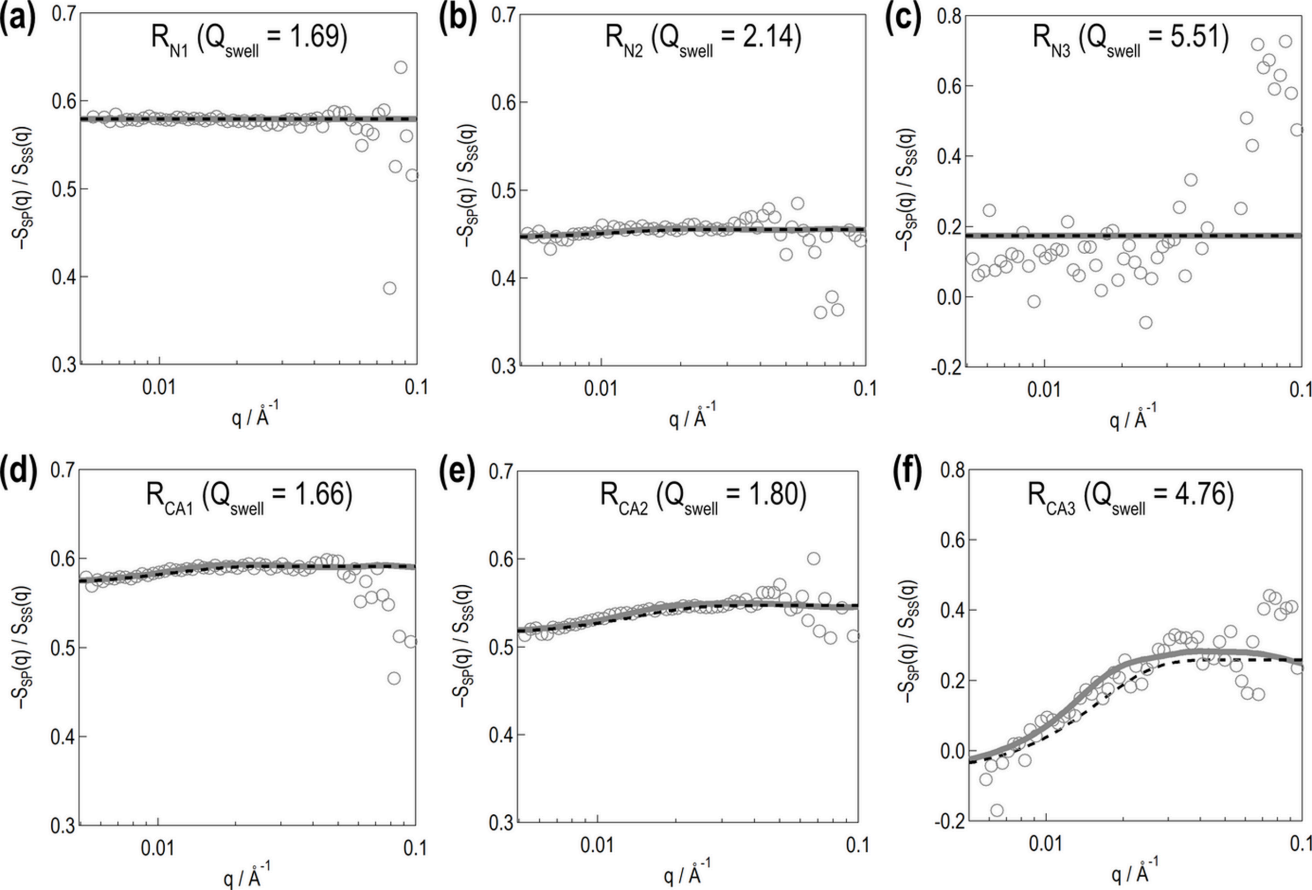
−*S*_SP_(*q*)/*S*_SS_(*q*) profiles. The top and bottom rows present the results for the rubber samples without and with the silane coupling agent, respectively. In each panel, the unfilled gray circles represent the experimentally obtained results, and the gray solid-line curves represent the profiles numerically calculated from the structure model. Here, the smear effect is considered. The black dashed-line curves indicate the numerical calculation results obtained using the approximate equation (38).

Fig. 8 ▸ shows the calculated −*S*_SP_(*q*)/*S*_SS_(*q*), with *R*_N1_ exhibiting a flat profile [Fig. 8 ▸(*a*)]. This indicates that the polymer and d-toluene are mixed homogeneously outside the silica. *R*_N2_ exhibits a slight depression at low *q*, indicating the formation of the polymer adsorption layer [Fig. 8 ▸(*b*)]. As the d-toluene volume fraction increases, the high-*q* constant value falls. As for *R*_N3_, as the amount of d-toluene increases to full swelling, the polymer fraction falls further [Fig. 8 ▸(*c*)]. No clear depression is identified, partially due to the larger fluctuations compared with those in the case of *R*_N1_ and *R*_N2_.

As shown in Figs. 8 ▸(*d*) and 8 ▸(*e*), *R*_CA1_ and *R*_CA2_ exhibit slight depressions at low *q*. As the d-toluene volume fraction increases, the high-*q* constant value falls and the low-*q* depression becomes clearer. As for *R*_CA3_, when the d-toluene volume fraction increases to full swelling, a significant low-*q* depression emerges [Fig. 8 ▸(*f*)]. The validity of the approximate equation (38) is discussed in Section 3.6.

### 3.4. Numerical calculation based on sphere collection model

In previous contrast-variation studies on swollen filler–rubber systems (Takenaka *et al.*, 2009 ▸; Takenaka *et al.*, 2012 ▸; Liu *et al.*, 2017 ▸), researchers calculated 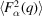 and 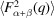 from decomposed PSFs and then applied the Beaucage unified equation (Beaucage & Schaefer, 1994 ▸; Beaucage, 1995 ▸; Beaucage, 1996 ▸; Beaucage, 2004 ▸) to obtain the structural parameters of the filler aggregate and adsorption layer. The Beaucage unified equation is advantageous for investigating filler aggregate systems with multilevel hierarchies. However, this approach does not provide an explicit formula for the cross term 〈*F*_α_(*q*)*F*_α+β_(*q*)〉. Therefore, many trials are required to determine the optimal parameter set.

Another traditional approach numerically calculates scattering profiles based on structure models. Nakanishi *et al.* (2024 ▸) developed a structure model that considers the formation of dimers, in which two primary particles contact each other. This approach was effective for their samples, which consisted of large silica particles with a radius of 523 Å. However, our samples, which were composed of silica particles with a radius of 105 Å, were likely to form aggregates composed of more than two primary particles. Hence, we built a structure model considering aggregates formed by numerous primary particles as follows.

**Figure 9:**
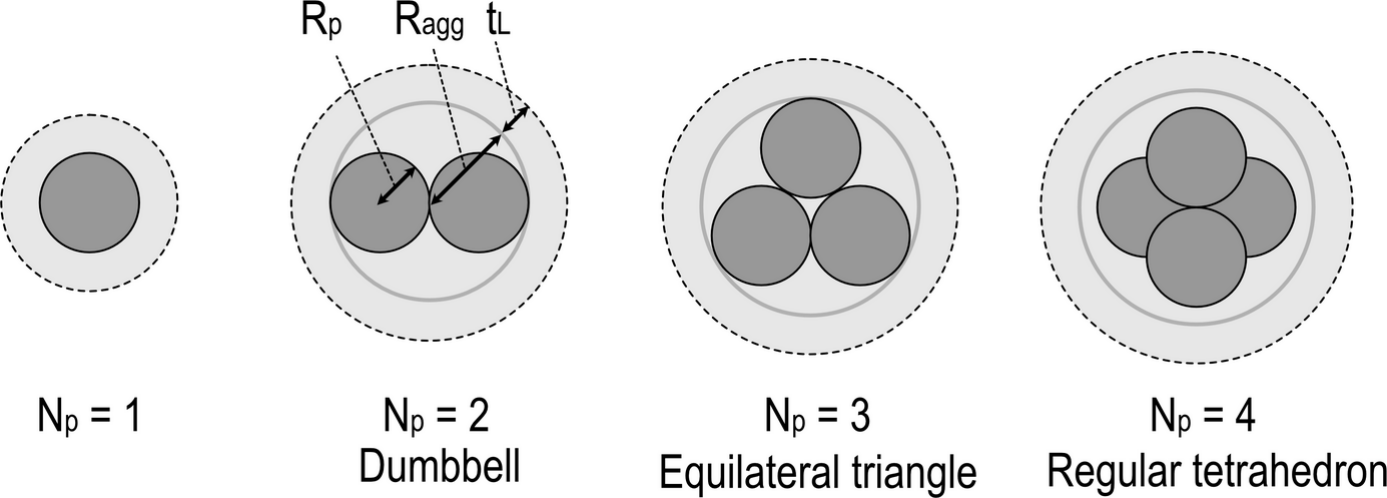
Schematic diagram of the sphere collection model. The particle configuration up to *N*_p_ = 4 is indicated.

In this structure model, silica aggregates are modeled as a collection of spherical particles, and the outer envelope of the polymer adsorption layer is modeled as a large sphere. Although this structure model is designed to reflect actual silica aggregates as much as possible, simplifications are made to reduce calculation difficulty. The structure model is defined by the following rules:

(i) In one aggregate, all primary particles have a common radius (*R*_p_).

(ii) Each particle center is placed at a lattice point of a face-centered cubic lattice. The lattice constant is set to 2
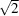
*R*_p_ so that particles at neighboring lattice points contact each other.

(iii) Among the possible aggregate configurations formed by *N*_p_ particles, we use only the ‘most compact’ one, as determined by the following procedure. The most compact configuration has the largest number of particle pairs with the shortest distance (2*R*_p_). If several configurations satisfy this criterion, then the numbers of particle pairs with the second-shortest distance are compared. If several configurations fulfill this requirement, then the numbers of particle pairs with the third-shortest distance are compared, and so on. Through this procedure, we manually determine the most compact configuration up to *N*_p_ = 19. As expected from these criteria, symmetric configuration is advantageous. We adopt a dumbbell for *N*_p_ = 2, an equilateral triangle for *N*_p_ = 3, a regular tetrahedron for *N*_p_ = 4, a regular octahedron for *N*_p_ = 6 and a cuboctahedron (including one particle at the center) for *N*_p_ = 13. Fig. 9 ▸ shows schematic diagrams of configurations up to *N*_p_ = 4. For *N*_p_ values not mentioned above, less symmetrical configurations are determined manually on the basis of the defined criteria. In the search process, we start from highly symmetrical configurations and then manually add or remove particles one by one.

(iv) The sum region of the silica aggregate and polymer adsorption layer (α+β) is defined as a single large sphere whose center coincides with the aggregate mass center. The radius of this sum region (*L*) is
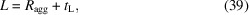
where *R*_agg_ is the circumsphere radius of the aggregate and *t*_L_ is the thickness of the polymer adsorption layer. In Fig. 9 ▸, the aggregate circumsphere and the outer envelope of the polymer adsorption layer are schematically indicated by the gray solid-line curves and black dashed-line curves, respectively.

(v) To account for aggregate polydispersity, we assume the distribution functions *W*_Np_(*N*_p_) and *W*_Rp_(*R*_p_). *W*_Np_(*N*_p_) is assumed to be a log-normal distribution,
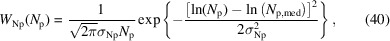
where *N*_p,med_ is the median value of *N*_p_ and σ_Np_ is the standard deviation of ln(*N*_p_). *W*_Rp_(*R*_p_) is assumed to be a Gaussian distribution:
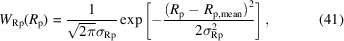
where *R*_p,mean_ is the mean value of *R*_p_ and σ_Rp_ is the standard deviation of *R*_p_.

Thus, the structure model for the silica aggregate and polymer adsorption layer is built. The aggregate configuration is defined in full detail for scattering intensity calculation. However, the adopted configuration is merely a typical one. A practical sample has various configurations that differ from the analyzed one. These slightly different configurations cannot be distinguished, as their subtle difference causes only a slight fluctuation in scattering profile, which is concealed by the effect of polydispersity in practical samples.

For the determined set of *N*_p_ particle center positions (**c**_*i*,Np_, where *i* = 1, 2,…, *N*_p_), the spatial distribution functions for regions α and α+β are, respectively,
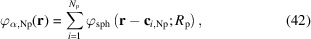

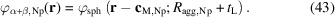
Here **c**_M,Np_ is the mass center position of an aggregate formed by *N*_p_ particles, which is calculated as
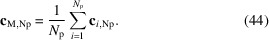
φ_sph_(**r**; *R*) is the spatial distribution of a sphere and defined as
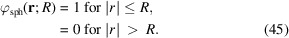
The form factors for φ_α,Np_(**r**) and φ_α+β,Np_(**r**) are
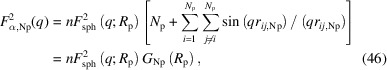

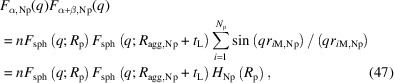

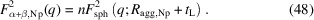
Here *n* is the number density of the aggregate [= (*f*_silica_/*Q*_swell_)/〈*V*_α_〉, where *f*_silica_ is the silica volume fraction]. *r*_*ij*,Np_ is the distance between particles *i* and *j* for the aggregate formed by *N*_p_ particles (= |**c**_*i*,Np_ − **c**_*j*,Np_|). *r*_*i*M,Np_ is the distance between particle *i* and the aggregate mass center for the aggregate formed by *N*_p_ particles (= |**c**_*i*,Np_ − **c**_M,Np_|). *F*_sph_(*q*; *R*) is the form amplitude of a sphere of radius *R*,
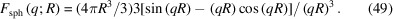
*G*_Np_(*R*_p_) in equation (46) is the interference term for the particle pairs in the aggregate. Possible *r*_*ij*,Np_ values are 2*R*_p_, 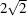
*R*_p_, 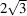
*R*_p_, 4*R*_p_ and so on, since the structure model assumes that the particle centers are located in a face-centered cubic lattice with the lattice constant of 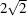
*R*_p_. For reducing the calculation effort, we rearranged *G*_Np_(*R*_p_) as
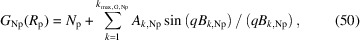
where *B*_*k*,Np_ is defined for sorting the *r*_*ij*,Np_ values and *A*_*k*,Np_ is the occurrence number of *B*_*k*,Np_ during the double summation in equation (46). *A*_*k*,Np_ and *B*_*k*,Np_ parameters calculated for the adopted *N*_p_ particle aggregate configuration are listed in Table 4 ▸.

*H*_Np_(*R*_p_) in equation (47) is the interference term between the particles and mass center of the aggregate. For reducing the calculation effort, we rearranged *H*_Np_(*R*_p_) as
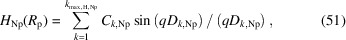
where *D*_*k*,Np_ is defined for sorting the *r*_*i*M,Np_ values and *C*_*k*,Np_ is the occurrence number of *D*_*k*,Np_ during the summation in equation (47). *C*_*k*,Np_ and *D*_*k*,Np_ parameters calculated for the adopted *N*_p_ particle aggregate configuration are listed in Table 5 ▸.

In addition, we calculate the circumsphere radius *R*_agg,Np_ of the adopted *N*_p_ particle aggregate configuration, and the obtained *R*_agg,Np_ values are listed in Table 6 ▸. In addition, the squared gyration radius 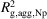 is calculated for the adopted *N*_p_ particle aggregate configuration:
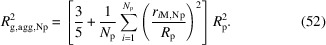
The obtained 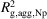 values are listed in Table 6 ▸.

We account for *N*_p_ and *R*_p_ polydispersity using the equations
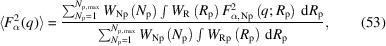

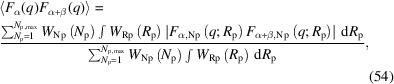

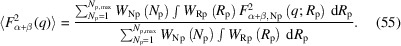


**Figure 10:**
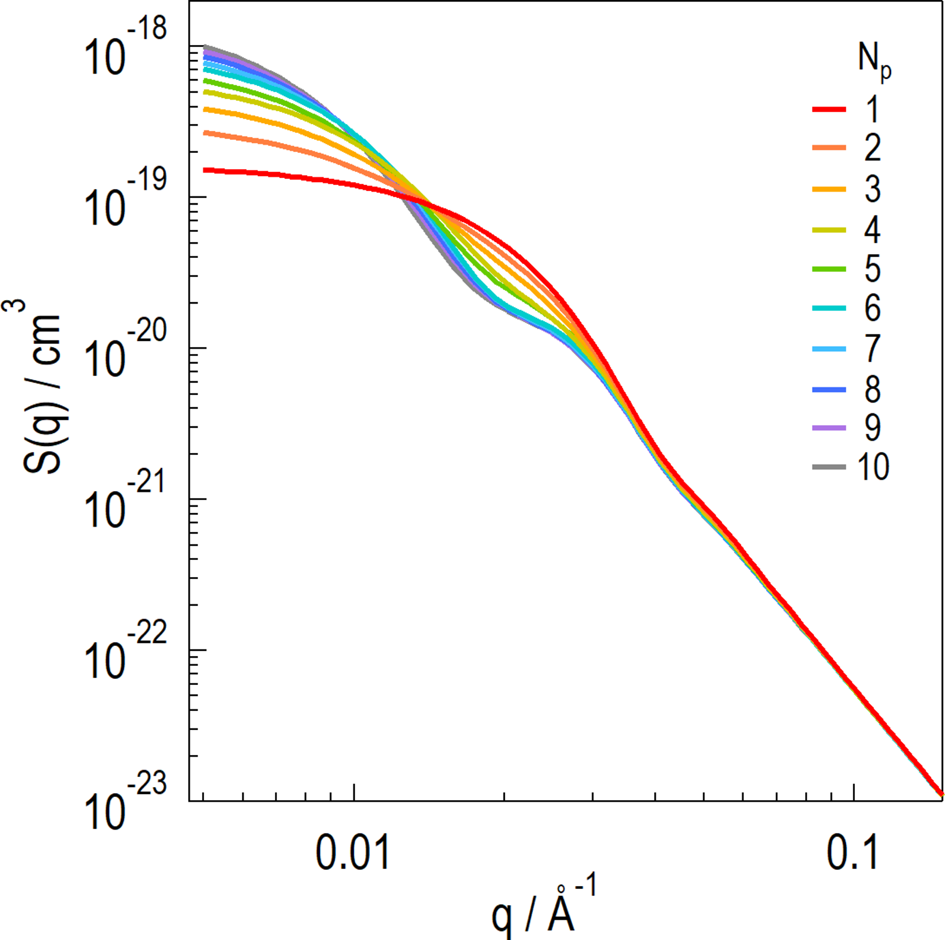
Numerically calculated profiles of silica aggregates formed by *N*_p_ particles [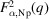] with a particle radius of *R*_p_ = 105 ± 25 Å (up to *N*_p_ = 10). The particle configuration of the silica aggregates was determined according to the criteria described in this article.

Fig. 10 ▸ shows the 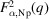 profiles numerically calculated using *R*_p_ = 105 ± 25 Å and *A*_*k*,Np_ and *B*_*k*,Np_ (Table 4 ▸). A profile with *N*_p_ = 1 indicates the form factor of a single sphere. The profile is flat at low *q* but follows *q*^−4^ at high *q*. The low-*q* scattering intensity increases with *N*_p_, whereas the high-*q* region of *q*^−4^ does not change. The shape of the shoulder at approximately *q* = 0.02 Å^−1^ gradually changes from convex to concave.

**Figure 11:**
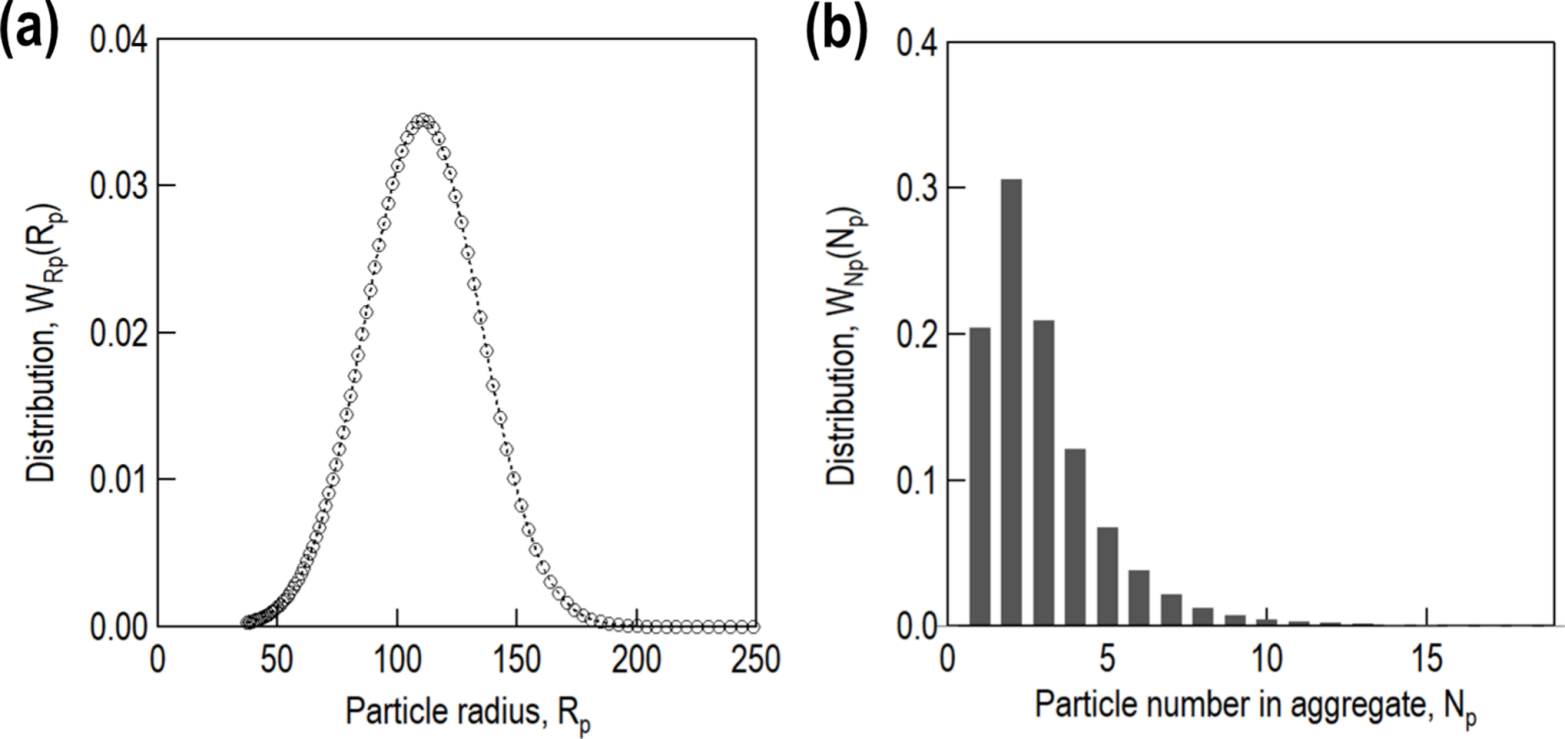
Distribution functions used for numerical calculation: *W*_Rp_(*R*_p_) (*a*) and *W*_Np_(*N*_p_) (*b*).

By considering *N*_p_ polydispersity, we calculate 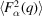, as indicated by the blue dotted-line curves in Fig. 6 ▸. The numerical calculations account for the smear effect of BL15 beam collimation (Takata *et al.*, 2015 ▸). This effect is only slight at low *q*. Consequently, the numerically calculated profiles excellently reproduce the experimentally obtained *S*_SS_(*q*) [= *n*
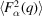
〈*K*(*q*)〉] despite the slight deviation at low *q* (*q* < 0.01 Å^−1^), which is probably due to 〈*K*(*q*)〉. The distribution functions *W*_Rp_(*R*_p_) and *W*_Np_(*N*_p_), which are used across all samples, are shown in Figs. 11 ▸(*a*) and 11 ▸(*b*), respectively. In a previous study (Nakanishi *et al.*, 2024 ▸), a silane coupling agent improved the dispersion of silica particles. However, such dispersion improvement was not observed in our samples.

**Figure 12:**
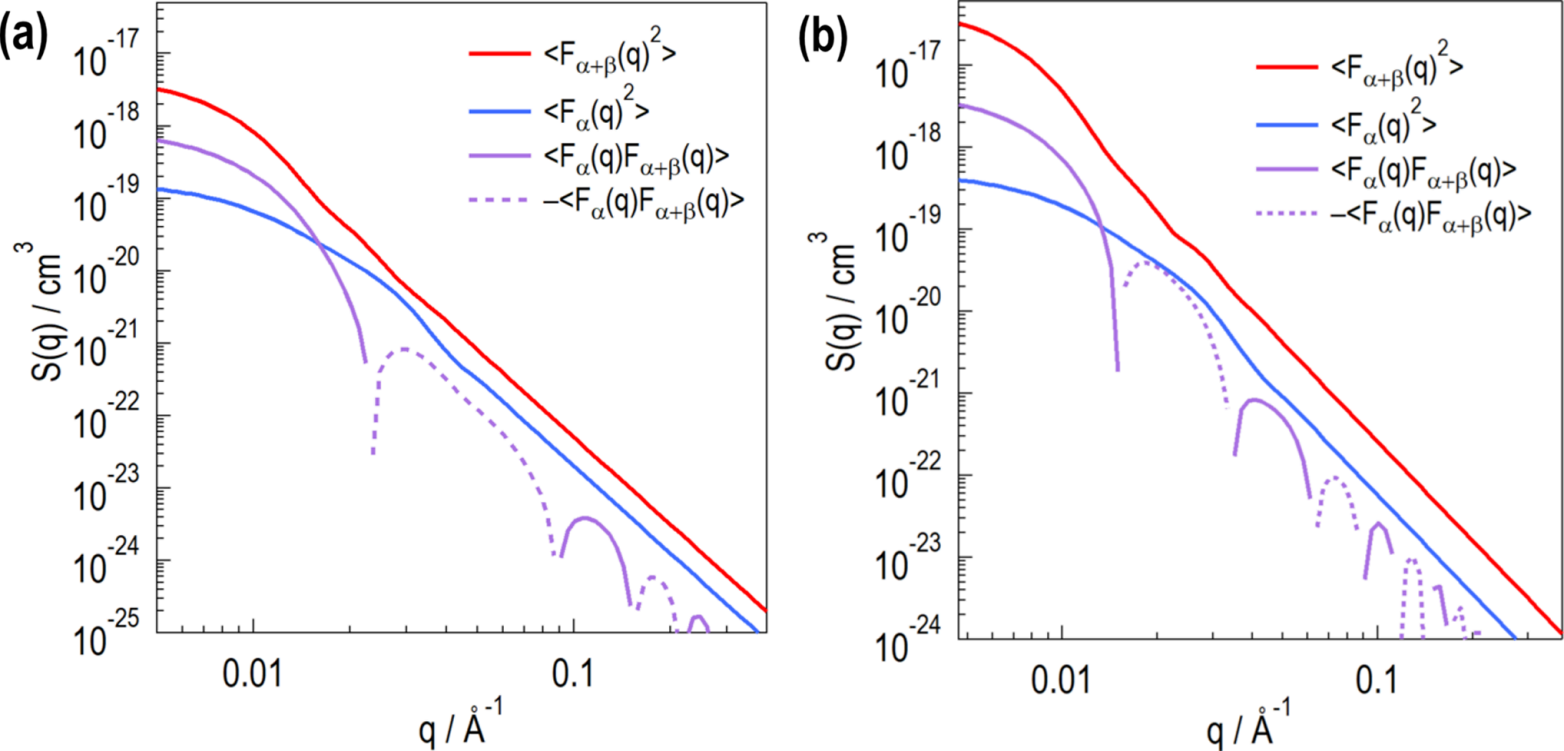
Numerically calculated 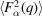, 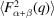 and 〈*F*_α_(*q*)*F*_α+β_(*q*)〉 for *t*_L_ = 50 Å (*a*) and *t*_L_ = 120 Å (*b*). For both panels, *R*_p_ = 105 ± 25 Å and the W_Rp_(*R*_p_) and W_Np_(N_p_) shown in Fig. 11 ▸ are used.

We evaluate *W*_Rp_(*R*_p_) and *W*_Np_(*N*_p_) by analyzing *S*_SS_(*q*). Next, we set *t*_L_ to calculate 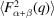 and 〈*F*_α_(*q*)*F*_α+β_(*q*)〉 (Fig. 12 ▸). The adopted *t*_L_ values [50 and 120 Å in Figs. 12 ▸(*a*) and 12 ▸(*b*), respectively] are optimized parameters for fitting the experimental results, as shown later in this section. In Fig. 12 ▸, 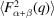 is always higher than 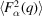. The *q*^−4^ slope of 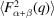 continues to lower *q* (*q* ∼ 0.01 Å^−1^) compared with that of 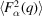. Fig. 12 ▸ also shows 〈*F*_α_(*q*)F_α+β_(*q*)〉. At low *q*, 〈*F*_α_(*q*)*F*_α+β_(*q*)〉 is between 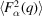 and 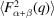. As *q* increases, 〈*F*_α_(*q*)*F*_α+β_(*q*)〉 decreases to a negative value more rapidly than 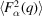 and then oscillates while decreasing in magnitude.

We determine φ_L_ and φ_M_, which are necessary for calculating *S*_PP_(*q*) and *S*_TT_(*q*) by equations (22) and (23), as follows. φ_L_ and φ_M_ are
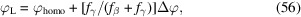

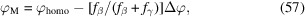
where φ_homo_ is given by (1 − *f*_silica_)/(*Q*_swell_ − *f*_silica_). *f*_silica_ is the silica volume fraction before swelling (= 0.05 in this study). *f*_α_, *f*_β_ and *f*_γ_, which are the volume fractions of regions α, β and γ, respectively, are
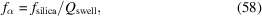

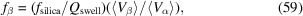

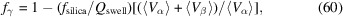
where 〈*V*_α_〉 and 〈*V*_β_〉 are the expected volumes of regions α and β, respectively, and can be calculated by the determined structure model. Therefore, only two parameters (*t*_L_ and Δφ) are adjustable in calculating *S*_PP_(*q*) and *S*_TT_(*q*). The *S*_PP_(*q*) and *S*_TT_(*q*) profiles with optimized parameters are drawn using green and orange dotted-line curves, respectively, in Fig. 6 ▸. At low *q*, the numerically calculated profiles deviate downwards similarly to *S*_SS_(*q*). This is because the numerical calculation does not consider the structure factor 〈*K*(*q*)〉 due to the higher-order structure. In Fig. 8 ▸, the numerically calculated ratio −*S*_SP_(*q*)/*S*_SS_(*q*) is indicated by the gray curves. With our use of this ratio, the effect of *K*(*q*) and *S*_G_(*q*) can be eliminated, enabling least-squares fitting in a wider *q* range to determine *t*_L_ and Δφ.

**Figure 13:**
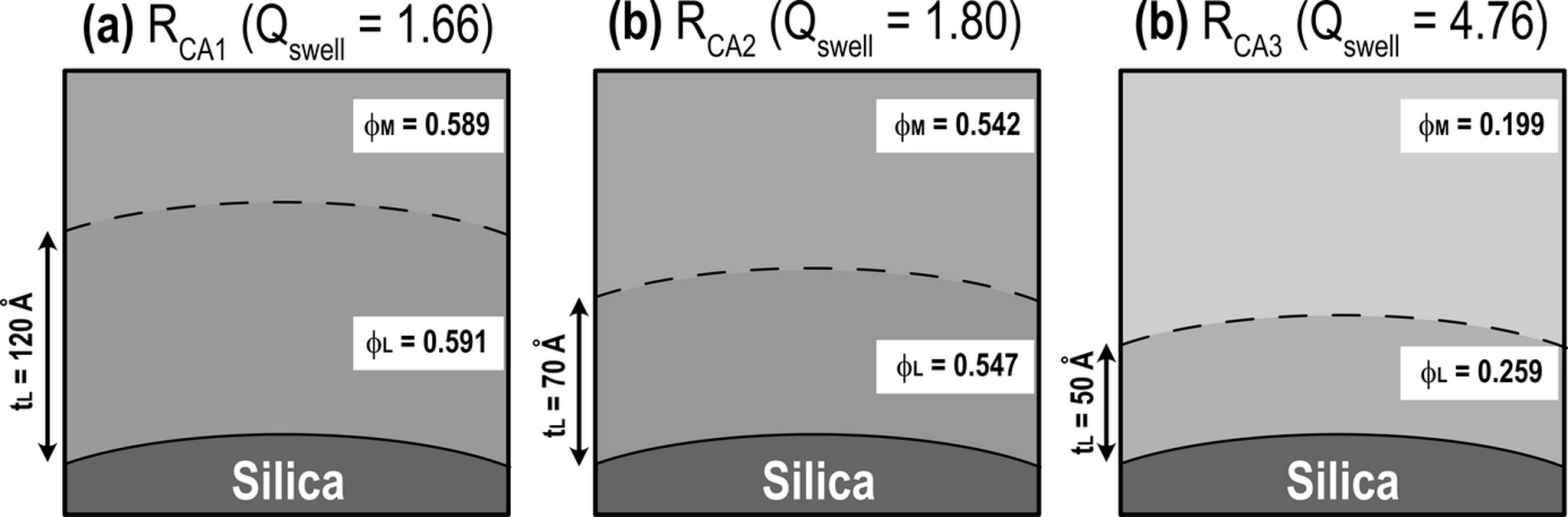
Schematic diagram of the polymer adsorption layer on the silica aggregate surface for R_CA1_ (*a*), R_CA2_ (*b*) and R_CA3_ (*c*). In each panel, the black dashed-line curve indicates the boundary between the polymer-dense layer and the surrounding matrix. The polymer chains are omitted for simplicity. Outside the silica, the background color indicates the polymer volume fraction.

The parameters determined by this fitting process are listed in Table 7 ▸. A schematic diagram of the microstructure around the silica particle surface of R_CA_ is shown in Fig. 13 ▸. As *Q*_swell_ increases, Δφ increases while *t*_L_ decreases. The volume of the silane coupling agent in one silica aggregate is 0.26 × 10^7^ Å^3^, as determined by the composition of the rubber ingredient, assuming that all silane coupling agents react with silica surfaces. This value is used across all R_CA_ samples. Moreover, the volume of polymer chains in the polymer adsorption layer is calculated as 〈*V*_β_〉/φ_L_. A comparison of these values suggests that the polymer adsorption layer contains not only the silane coupling agent but also polymer chains confined by the silane coupling agent. For R_CA1_, R_CA2_ and R_CA3_, the computed volume of polymer chains in the polymer adsorption layer is 43, 23 and 6.8 times larger than that of the silane coupling agent, respectively. The confinement degree is expected to decrease with distance from the silica aggregate surface. It was suggested that in the fully swollen state only tightly confined polymer chains are included in the polymer adsorption layer. By contrast, in a less swollen state, the polymer adsorption layer additionally contains moderately confined polymer chains. NMR relaxation times have shown that an intermediate region forms outside the confined polymer around filler particles (O’Brien *et al.*, 1976 ▸). The correlation between the results of these two approaches will further elucidate the structure of the polymer adsorption layer.

For R_N_, a slight indication of the formation of a polymer adsorption layer emerges in the less swollen state (R_N2_). In the fully swollen state (R_N3_), little indication of formation is found. Even for the sample without a silane coupling agent, a polymer adsorption layer, reflecting loose confinement around the silica aggregate, is observed. However, polymer adsorption layers reflecting tight confinement around silica aggregates are not observed naturally in samples without silane coupling agents.

### 3.5. Separation of scattering contribution due to polymer in d-toluene

*S*_G_(*q*), observed in swollen rubber samples, provides insights into polymer networks and cross-links (Karino *et al.*, 2007 ▸; Ikeda *et al.*, 2009 ▸; Suzuki *et al.*, 2010 ▸). For silica-filled swollen rubber, the OZ contribution at high *q* can be analyzed easily, as the scattering contributions of filler particles are insignificant in the high-*q* region. By contrast, the DB contribution at low *q* is difficult to analyze because the scattering contribution of filler particles is prominent in the low-*q* region. The contrast-variation approach provides a way to separate *S*_G_(*q*).

**Figure 14:**
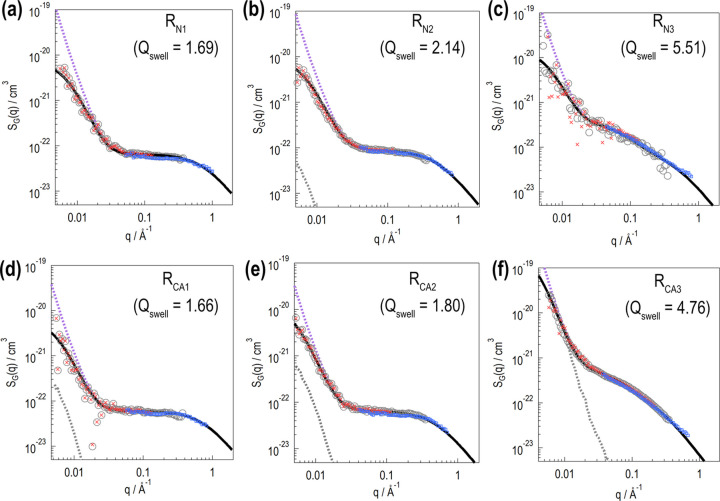
*S*_G_(*q*) profiles due to polymer chains in solvent. The top and bottom rows present the results for the rubber samples without and with the silane coupling agent, respectively. In each panel, the experimentally obtained *S*_G_(*q*) is shown by the unfilled gray circles. The *S*_G_(*q*) profiles numerically calculated using equations (30) and (63) are shown by the black solid-line and purple dotted-line curves, respectively. In panels (*b*), (*d*), (*e*) and (*f*), the experimentally obtained *n*Δφ^2^
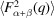
〈*K*(*q*)〉 contribution is shown by the gray dashed-line curve. The experimentally obtained profiles in the unpolarized state for compensation in the high-*q* region are shown by the blue squares. The *S*_G,approx_(*q*) numerically calculated using equation (65) is shown by the red crosses. In these numerical calculations, the smear effect is considered.

By substituting equations (21), (22) and (24) into equation (17), we obtain
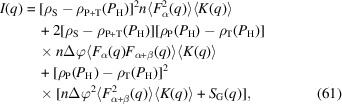
where ρ_P+T_ is the SLD of the matrix domain, assuming the matrix is homogeneous: ρ_P+T_ = φ_homo_ρ_P_ + (1 − φ_homo_)ρ_T_. *I*(*q*) consists of three terms with different *P*_H_ dependence. Both *n*Δφ^2^
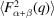
〈*K*(*q*)〉 and *S*_G_(*q*) are included in the final term. A set of three independent PSFs can be determined through contrast variation. From these PSFs, another set of three independent functions [*n*
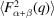
〈*K*(*q*)〉, *n*Δφ〈*F*_α+β_(*q*) × *F*_α+β_(*q*)〉〈*K*(*q*)〉 and *n*Δφ^2^
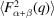
〈*K*(*q*)〉 + *S*_G_(*q*)] can be obtained. However, contrast variation itself cannot determine *n*Δφ^2^
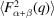
〈*K*(*q*)〉 and *S*_G_(*q*) separately. In cases with an insignificant Δφ (*e.g.* less swollen or homogeneously swollen states), *n*Δφ^2^
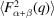
〈*K*(*q*)〉 can be neglected to determine *S*_G_(*q*) easily. However, in cases with a significant Δφ (*e.g.* R_CA3_), *S*_G_(*q*) is difficult to determine. In this case, *S*_G_(*q*) should be evaluated using the following equation, which is derived from equations (21), (22) and (24):
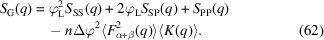
We determine *n*Δφ^2^
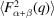
〈*K*(*q*)〉 through the structure model analysis in Section 3.4. The evaluated *S*_G_(*q*) profiles are shown by the unfilled gray circles in Fig. 14 ▸. Each panel shows a clear DB contribution at low *q*. However, the shape of the OZ contribution at high *q* is not completely covered, because of the limited *q* range of our setup. This is compensated for by overlaying the SANS profile obtained in the unpolarized state (*P*_H_ = 0.1%), as shown by the blue squares in Fig. 14 ▸. The low-*q* side of these unpolarized-state profiles is omitted, and the intensity shifts. These unpolarized-state profiles cover higher *q* values, as they involve full-range neutron wavelengths (1.0 < λ < 7.6 Å).

The profiles are fitted using equation (30), as indicated by the black solid-line curves. In addition, we numerically calculate profiles using the following equation, where the DB function is replaced with the power-law function (*q*^−4^) derived from Porod’s law:
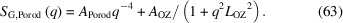
Here *A*_Porod_ = *A*_DB_/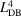. *S*_G,Porod_(*q*) is shown in Fig. 14 ▸ by the purple dotted-line curves. The experimentally obtained *S*_G_(*q*) is lower than *S*_G,Porod_(*q*) at low *q*. This indicates the validity of profile fitting using the DB function, although the observed *q* range does not cover the full shape of the DB function. The evaluated *A*_DB_, *L*_DB_ and *A*_Porod_ parameters are listed in Table 7 ▸.

For cases with polymer adsorption layer formation, we subtract the *n*Δφ^2^
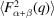
〈*K*(*q*)〉 term to assess *S*_G_(*q*) using equation (62). Here, we use the *n*Δφ^2^
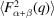
〈*K*(*q*)〉 value determined via structure model analysis. Fig. 14 ▸ shows *n*Δφ^2^
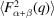
〈*K*(*q*)〉 as the gray dotted-line curves. For R_N2_, R_CA1_ and R_CA2_, the *n*Δφ^2^
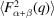
〈*K*(*q*)〉 contribution is much smaller than that of *S*_G_(*q*). For R_CA3_, which has a significant polymer adsorption layer, the calculated *S*_G_(*q*) is comparable to the *n*Δφ^2^
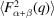
〈*K*(*q*)〉 contribution at low *q*. As mentioned above, *S*_G_(*q*) cannot be determined directly in this case. *S*_G_(*q*) can only be accurately evaluated when *n*Δφ^2^
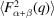
〈*K*(*q*)〉 is obtained through structure model analysis.

**Figure 15:**
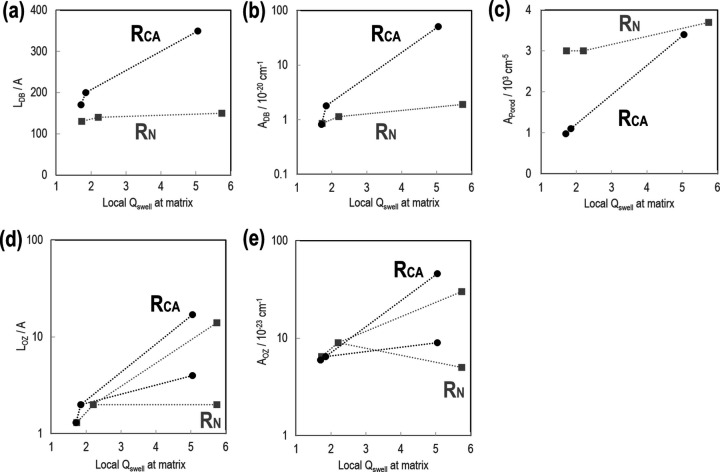
Parameters *L*_DB_ (*a*), *A*_DB_ (*b*), *A*_Porod_ (*c*), *L*_OZ_ (*d*) and *A*_OZ_ (*e*) as functions of local *Q*_swell_ in the matrix (= 1/φ_M_). In each panel, the black circles and gray squares indicate the results for R_CA_ and R_N_, respectively. The dotted lines are guides for the reader.

**Figure 16:**
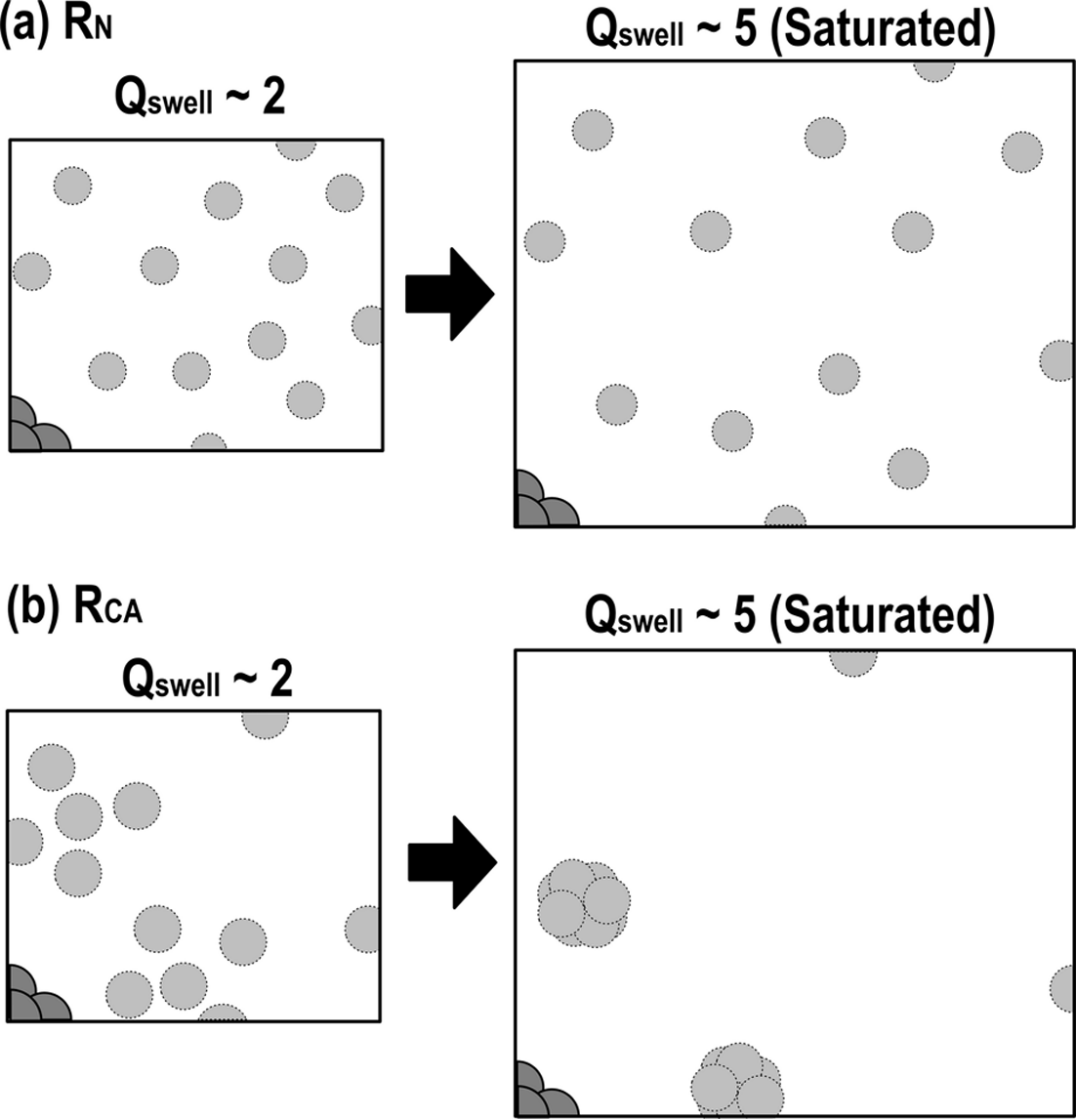
Schematic diagram of polymer-dense domain distribution for R_N_ (*a*) and R_CA_ (*b*). In each panel, the dark gray circles at the bottom-left corner indicate the silica aggregates, and the light gray circles indicate the polymer-dense domains. The polymer chains are omitted for simplicity.

Fig. 15 ▸ shows the determined parameters (*L*_DB_, *A*_DB_, *A*_Porod_, *L*_OZ_ and *A*_OZ_) as functions of the local *Q*_swell_ in the matrix (= 1/φ_M_). Concerning the low-*q* DB function, which is due to the inhomogeneous distribution of polymer chains, R_N_ exhibits almost constant *L*_DB_ and *A*_DB_ values, regardless of the swelling ratio. By contrast, R_CA_ shows significant increases in *L*_DB_ and *A*_DB_ with an increase in the swelling ratio. This result is ascribed to the spatial distribution shown in Fig. 16 ▸. For R_N_, the polymer-dense domains are distributed sparsely. For R_CA_, the polymer-dense domains are distributed mostly near silica aggregates. With an increase in solvent fraction, polymer-dense domains enlarge. By increasing *Q*_swell_ from 1.66 to 4.76, we find that *L*_DB_ increases by a factor of 2.1 (= 350/170) and *A*_DB_ increases by a factor of 62 (= 51/0.82). This finding is explained quantitatively as follows. *A*_DB_ is proportional to *n*_PD_Δ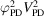, where *n*_PD_ is the number of polymer-dense regions per volume, Δφ_PD_ is the difference in polymer volume fraction between a polymer-dense region and the surrounding matrix, and *V*_PD_ is the volume of a polymer-dense domain. Because of the 2.1-times increase in *L*_DB_, *V*_PD_ should increase by a factor of 8.7 [= (350/170)^3^]. Approximately nine polymer-dense domains in less swollen states merge to create larger domains in the fully swollen state. Furthermore, *n*_PD_ should decrease by a factor of 1/25.5 {= 1/[(350/170)^3^ × (1.69/4.96)]}, considering the swelling-induced volume increase. Δ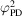 should increase by a factor of 21 (= 62 × 25.5/8.7^2^) to reproduce the obtained *A*_DB_. The polymer volume fraction in the matrix can be roughly estimated via (*Q*_swell_ − *f*_silica_)/(1 − *f*_silica_) to be 0.59 and 0.20 at *Q*_swell_ = 1.66 and *Q*_swell_ = 4.76, respectively. Assuming that the volume fraction of the polymer-dense layer is 0.7 and is not significantly affected by the swelling ratio, Δ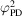 should increase by a factor of 21 to reproduce the observed results. Although the volume fraction of the polymer-dense layer may be affected by the swelling ratio, this microscopic picture roughly reproduces the silica-filled rubber sample containing the silane coupling agent.

The obtained microscopic picture [Fig. 16 ▸(*b*)] may be a result of the presumed chemical process during the vulcanization of R_CA_ as follows. Silane coupling agents have silanol groups for binding silica particle surfaces, and polysulfide bonds for creating cross-links with surrounding polymers. Therefore, for R_CA_, polysulfide bonds generate sulfur radicals concentrated around the silica aggregates, creating nearby cross-links. This can explain the formation of polymer-dense regions [Fig. 16 ▸(*b*)].

Two OZ functions are used for both R_CA3_ and R_N3_ at high *q*. Two sets of *A*_OZ_ and *L*_OZ_ are determined and they both increase with *Q*_swell_. It is natural that the polymer mesh size increases with increasing amount of solvent in the polymer. In the fully swollen state, R_CA_ exhibits larger *A*_OZ_ and *L*_OZ_ than R_N_. Therefore, R_CA_ has a sparser cross-link distribution in the matrix than R_N_. For R_CA_, the merging of polymer-dense domains at the fully swollen state, as discussed in the DB function results, could lead to higher dilution in the matrix.

Nakanishi *et al.* (2024 ▸) significantly increased *A*_DB_ by introducing an excessive amount of a silane coupling agent during sample preparation. They attributed the large observed *A*_DB_ to the cross-link inhomogeneity formed by the excess silane coupling agent separate from the silica aggregates. By contrast, we used a much smaller amount of silane coupling agent.

### 3.6. Further discussion on the model-free approach based on the Guinier approximation

As stated in Section 3.3, we use the Guinier approximation [equations (35)–(38)]. The −*S*_SP_(*q*)/*S*_SS_(*q*) value numerically calculated using equation (38) is shown by the black dashed-line curve in Fig. 8 ▸. It is close to the results calculated through structure model analysis, as shown by the gray solid-line curve in Fig. 8 ▸, although a slight deviation is found in the fully swollen case (R_CA3_). The approximate equation is useful for rough estimation. Here, we extend this approach further to *S*_G_(*q*) estimation.

From equations (35)–(37), we obtain the approximate equation
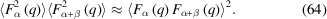
By applying this to equations (21), (22), (24) and (62), we develop another approach to approximating *S*_G_(*q*):
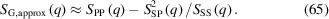
However, this equation is only satisfied at low *q*. Nonetheless, a benefit of this approach is that it eliminates the need to evaluate the *n*Δφ^2^
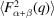
〈*K*(*q*)〉 contribution separately. As shown by the red crosses in Fig. 14 ▸, the calculated *S*_G,approx_(*q*) values for all samples coincide well with the *S*_G_(*q*) values obtained in the previous section. The *S*_G,approx_(*q*) value computed using equation (65) is useful for DB-term parameter evaluation. This approach can avoid the need for numerical calculation based on the sphere collection model. As mentioned earlier, the DB term is difficult to assess accurately because significant scattering due to filler particles overlaps at low *q*.

The difference between *S*_G,approx_(*q*) and *S*_G_(*q*) is
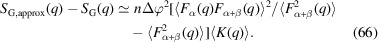
Hence, if equation (64) is satisfied, then *S*_G,approx_(*q*) becomes equal to *S*_G_(*q*). The difference is proportional to Δφ^2^. Hence, *S*_G,approx_(*q*) and *S*_G_(*q*) should coincide well for less swollen or homogeneously swollen states (Δφ << 1).

**Figure 17:**
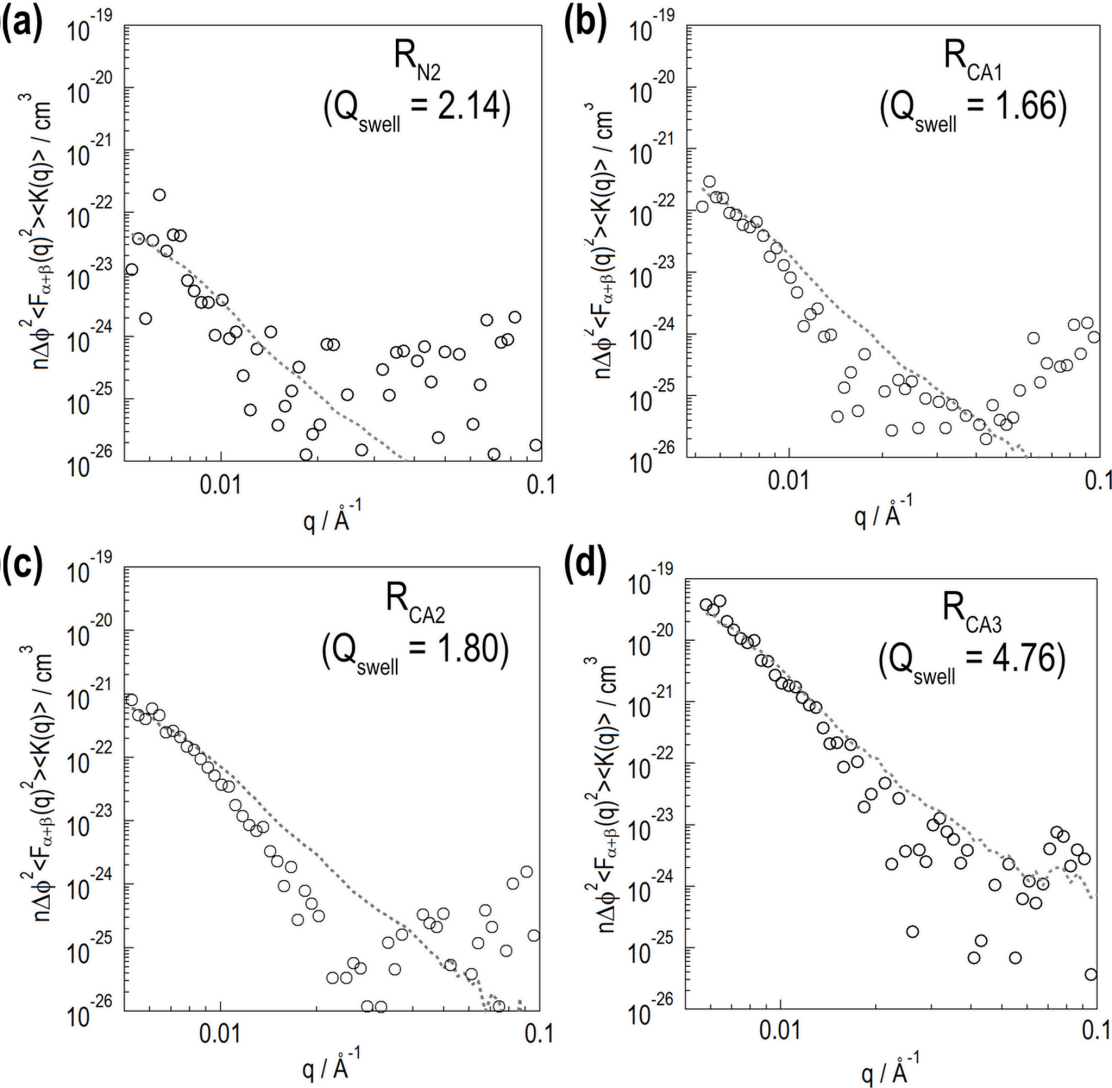
*n*Δφ^2^
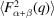
〈*K*(*q*)〉 profiles. In each panel, the gray dashed-line curves indicate the *n*Δφ^2^
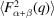
〈*K*(*q*)〉 profiles obtained through numerical cal­cula­tion based on the sphere collection model. The unfilled black circles indicate the *n*Δφ^2^
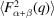
〈*K*(*q*)〉 contribution obtained using the approximate equation (67). The profiles obtained by the two approaches coincide well at low *q* but deviate at high *q*.

On the basis of this approximation, an equation that yields *n*Δφ^2^
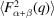
〈*K*(*q*)〉 directly from PSFs can be derived as
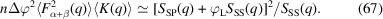
The *n*Δφ^2^
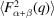
〈*K*(*q*)〉 contribution obtained from this approximate equation is indicated by the unfilled black circles in Fig. 17 ▸. The *n*Δφ^2^
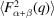
〈*K*(*q*)〉 contribution numerically calculated from the sphere collection model is indicated by gray dashed-line curves. The curves coincide well at low *q*. However, the profile obtained by the approximate equation deviates downwards as *q* increases, as the Guinier approximation is only valid at low *q*.

## 4. Conclusion

Spin-contrast-variation SANS was applied to silica-filled rubber with and without a silane coupling agent in partially and fully swollen states. SANS profiles were obtained at various *P*_H_ using a DNP cryostat (1.2 K and 3.35 T). Each sample was regarded as a system composed of silica, polymer and d-toluene components, and the PSF of each component was evaluated. Analytically, −*S*_SP_(*q*)/*S*_SS_(*q*) is proposed as a useful indicator, providing the polymer volume fraction (φ_L_) as a flat region at high *q* and as a depression at low *q* in the presence of a polymer adsorption layer. Furthermore, we built a sphere collection model for silica aggregates and the surrounding polymer adsorption layer. Although this model includes simplifications for reduced calculation effort, the obtained profiles excellently reproduced the experimental results. Consequently, the radius distribution of the primary silica particles and the particle number distribution in a silica aggregate were determined. Furthermore, on the basis of this structure model, *t*_L_ and Δφ at various *Q*_swell_ were calculated. For R_CA_, *t*_L_ decreased and Δφ increased as *Q*_swell_ increased. Based on the structural parameters, the computed ratios of the polymer volume in the polymer adsorption layer to the volume of the silane coupling agent were 43, 23 and 6.8 for R_CA_ at *Q*_swell_ values of 1.7, 1.8 and 4.8, respectively. In the fully swollen state, the polymer adsorption layer contained only tightly confined polymer chains. In a less swollen state, it additionally contained moderately confined polymer chains. Furthermore, the *S*_G_(*q*) contribution was accurately evaluated. The obtained *S*_G_(*q*) profiles indicated a clear difference between R_N_ and R_CA_. R_CA_ formed polymer-dense domains distributed mostly around the silica aggregates. As *Q*_swell_ increased, the polymer-dense domains merged to create larger domains. By contrast, R_N_ formed sparsely distributed polymer-dense domains. In addition, on the basis of the Guinier approximation (satisfied only at low *q*), we derived several approximate equations. With these approximate equations, rough estimates could be obtained without requiring structure-model-based analysis. The validity of the approximate equations was investigated using the present study results. Finally, we found reasonable alignment between these approaches. The developed analytical approaches can be used not only in spin-contrast-variation SANS studies but also in conventional contrast-variation ones.

## Supplementary Material

Table S1 List of the symbols used in this study. DOI: 10.1107/S1600576726000361/ge5181sup1.pdf

## Figures and Tables

**Table 1:** Composition (vol.%) of rubber samples before swelling

Sample	S-SBR (VSL4720)	Silica (VN3)	Silane coupling agent (Si266)	Sulfur	Accelerator (TBBS)	Accelerator (DPG)
R_N_	92.950	5.014	0.0	0.636	0.681	0.720
R_CA_	92.230	4.975	0.774	0.631	0.675	0.715

**Table 2:** Calculated scattering length density and total and incoherent scattering cross sections for each ingredient

Ingredient	SLD (×10^10^ cm^−2^)	Σ_tot_ (cm^−1^)	Σ_inc_ (cm^−1^)
SBR (VSL4720)	0.59 + 8.44*P*_H_	5.00 − 3.89*P*_H_	1.52(3 − 2*P*_H_ − 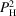 )
Silica (VN3)	3.08	0.208	0.000
Silane coupling agent (Si266)	0.15 + 7.91*P*_H_	4.63 − 3.64*P*_H_	1.45(3 − 2*P*_H_ − 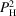 )
Sulfur	1.08	0.059	0.000
Accelerator (TBBS)	1.45 + 6.54*P*_H_	3.98 − 3.01*P*_H_	1.20(3 − 2*P*_H_ − 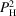 )
Accelerator (DPG)	2.26 + 6.48*P*_H_	4.03 − 2.98*P*_H_	1.19(3 − 2*P*_H_ − 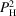 )
Deuterated toluene (99 at.% D)	5.62 + 0.06*P*_H_ + 1.22*P*_D_	0.60 − 0.03*P*_H_ + 0.17*P*_D_*P*_N_	0.012(3 − 2*P*_H_ − 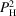 ) + 0.046(2 − *P*_D_ − 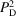 )
TEMPO	0.27 + 9.19*P*_H_	5.41 − 4.23*P*_H_	1.68(3 − 2*P*_H_ − 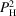 )

**Table 3:** Calculated total and incoherent scattering cross sections for each sample

Sample	*Q*_swell_	Thickness (cm)	Σ_tot_ (cm^−1^)	Σ_inc_ (cm^−1^)
R_N1_	1.69	0.079	3.07 − 2.21*P*_H_ + 0.07*P*_D_	0.864(3 − 2*P*_H_ − 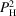 ) + 0.018(2 − *P*_D_ − 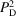 )
R_N2_	2.14	0.098	2.55 − 1.75*P*_H_ + 0.09*P*_D_	0.686(3 − 2*P*_H_ − 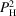 ) + 0.024(2 − *P*_D_ − 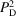 )
R_N3_	5.51	0.250	1.38 − 0.72*P*_H_ + 0.14*P*_D_	0.281(3 − 2*P*_H_ − 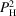 ) + 0.037(2 − *P*_D_ − 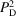 )
R_CA1_	1.66	0.100	3.11 − 2.25*P*_H_ + 0.07*P*_D_	0.879(3 − 2*P*_H_ − 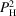 ) + 0.018(2 − *P*_D_ − 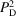 )
R_CA2_	1.80	0.127	2.91 − 2.07*P*_H_ + 0.07*P*_D_	0.809(3 − 2*P*_H_ − 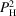 ) + 0.020(2 − *P*_D_ − 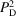 )
R_CA3_	4.76	0.125	1.49 − 0.82*P*_H_ + 0.13*P*_D_	0.321(3 − 2*P*_H_ − 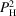 ) + 0.036(2 − *P*_D_ − 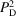 )

**Table 4:** Parameters *A*_*k*,Np_ (upper values in each cell) and *B*_*k*,Np_ (lower values in each cell) calculated for the adopted *N*_p_ particle aggregate configuration

*N*_p_ \ *k*	1	2	3	4	5	6
2	2					
	2*R*_p_					
3	6					
	2*R*_p_					
4	12					
	2*R*_p_					
5	16	4				
	2*R*_p_	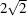 *R*_p_				
6	24	6				
	2*R*_p_	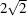 *R*_p_				
7	30	6	6			
	2*R*_p_	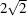 *R*_p_	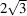 *R*_p_			
8	36	8	12			
	2*R*_p_	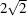 *R*_p_	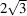 *R*_p_			
9	42	10	18	2		
	2*R*_p_	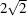 *R*_p_	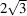 *R*_p_	4*R*_p_		
10	48	14	24	4		
	2*R*_p_	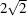 *R*_p_	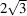 *R*_p_	4*R*_p_		
11	54	16	32	8		
	2*R*_p_	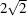 *R*_p_	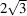 *R*_p_	4*R*_p_		
12	62	20	40	10		
	2*R*_p_	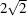 *R*_p_	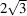 *R*_p_	4*R*_p_		
13	72	24	48	12		
	2*R*_p_	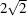 *R*_p_	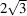 *R*_p_	4*R*_p_		
14	80	26	56	12	8	
	2*R*_p_	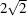 *R*_p_	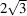 *R*_p_	4*R*_p_	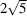 *R*_p_	
15	72	24	72	18	24	
	2*R*_p_	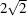 *R*_p_	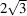 *R*_p_	4*R*_p_	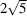 *R*_p_	
16	96	30	72	18	24	
	2*R*_p_	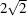 *R*_p_	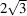 *R*_p_	4*R*_p_	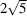 *R*_p_	
17	104	32	80	22	32	2
	2*R*_p_	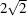 *R*_p_	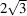 *R*_p_	4*R*_p_	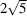 *R*_p_	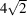 *R*_p_
18	112	34	88	28	40	4
	2*R*_p_	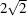 *R*_p_	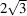 *R*_p_	4*R*_p_	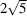 *R*_p_	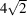 *R*_p_
19	120	36	96	36	48	6
	2*R*_p_	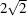 *R*_p_	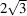 *R*_p_	4*R*_p_	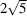 *R*_p_	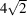 *R*_p_

**Table 5:** Parameters *C*_*k*,Np_ (upper values in each cell) and *D*_*k*,Np_ (lower values in each cell) calculated for the adopted *N*_p_ particle aggregate configuration

*N*_p_ \ *k*	1	2	3	4	5	6	7	8
1	1							
	0.000							
2	2							
	1.000*R*_p_							
3	3							
	1.155*R*_p_							
4	4							
	1.225*R*_p_							
5	1	4						
	1.131*R*_p_	1.442*R*_p_						
6	6							
	1.414*R*_p_							
7	3	3	1					
	1.245*R*_p_	1.641*R*_p_	2.100*R*_p_					
8	2	2	2	2				
	1.118*R*_p_	1.500*R*_p_	1.803*R*_p_	2.062*R*_p_				
9	1	2	2	1	1	2		
	0.969*R*_p_	1.352*R*_p_	1.648*R*_p_	1.899*R*_p_	2.012*R*_p_	2.222*R*_p_		
10	1	4	1	4				
	0.849*R*_p_	1.523*R*_p_	1.980*R*_p_	2.173*R*_p_				
11	1	2	1	2	2	2	1	
	0.315*R*_p_	1.734*R*_p_	1.836*R*_p_	1.933*R*_p_	2.025*R*_p_	2.112*R*_p_	2.197*R*_p_	
12	1	1	4	2	4			
	0.167*R*_p_	1.833*R*_p_	1.922*R*_p_	2.007*R*_p_	2.088*R*_p_			
13	1	12						
	0.000	2.000*R*_p_						
14	1	4	4	4	1			
	0.202*R*_p_	1.863*R*_p_	2.010*R*_p_	2.148*R*_p_	2.626*R*_p_			
15	1	1	4	2	4	1	2	
	0.267*R*_p_	1.733*R*_p_	1.881*R*_p_	2.018*R*_p_	2.146*R*_p_	2.267*R*_p_	2.647*R*_p_	
16	1	3	6	3	3			
	0.306*R*_p_	1.759*R*_p_	2.023*R*_p_	2.257*R*_p_	2.663*R*_p_			
17	1	1	4	2	4	1	2	2
	0.235*R*_p_	1.764*R*_p_	1.893*R*_p_	2.013*R*_p_	2.127*R*_p_	2.235*R*_p_	2.667*R*_p_	2.838*R*_p_
18	1	4	4	4	1	4		
	0.157*R*_p_	1.892*R*_p_	2.006*R*_p_	2.114*R*_p_	2.671*R*_p_	2.833*R*_p_		
19	1	12	6					
	0.000	2.000*R*_p_	2.828*R*_p_					

**Table 6:** Parameters *R*_agg,Np_ and 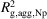 calculated for the adopted *N*_p_ particle aggregate configuration

N_p_	R_agg,Np_	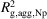
1	1.000*R*_p_	0.600 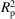
2	2.000*R*_p_	1.600 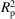
3	2.155*R*_p_	1.933 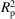
4	2.225*R*_p_	2.100 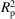
5	2.442*R*_p_	2.520 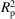
6	2.414*R*_p_	2.600 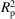
7	3.100*R*_p_	3.049 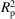
8	3.062*R*_p_	3.350 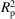
9	3.222*R*_p_	3.662 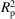
10	3.173*R*_p_	3.880 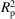
11	3.197*R*_p_	4.137 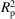
12	3.088*R*_p_	4.239 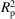
13	3.000*R*_p_	4.292 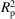
14	3.626*R*_p_	4.559 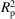
15	3.653*R*_p_	4.796 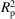
16	3.663*R*_p_	5.006 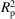
17	3.838*R*_p_	5.251 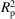
18	3.833*R*_p_	5.464 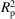
19	3.828*R*_p_	5.653 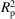

**Table 7:** Structural parameters used for the sphere collection model

	R_N1_	R_N2_	R_N3_		R_CA1_	R_CA2_	R_CA3_	
*Q*_swell_	1.69	2.14	5.51		1.66	1.80	4.76	
φ_homo_	0.579	0.4545	0.174		0.590	0.543	0.202	
φ_L_	0.579	0.4553	0.174		0.591	0.547	0.259	
φ_M_	0.579	0.4543	0.174		0.589	0.542	0.199	
Δφ = (φ_L_− φ_M_)	0.000	0.001	0.000		0.002	0.005	0.060	
*t*_L_ (Å)	–	120	–		120	70	50	
*R*_p,mean_ (Å)	105	105	105		105	105	105	
σ_Rp_ (Å)	25	25	25		25	25	25	
*N*_p,med_	2.5	2.5	2.5		2.5	2.5	2.5	
σ_Np_	0.6	0.6	0.6		0.6	0.6	0.6	
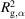 (10^4^ Å^2^)	2.02	2.02	2.02		2.02	2.02	2.02	
〈*V*_α_〉 (10^7^ Å^3^)	1.68	1.68	1.68		1.68	1.68	1.68	
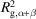 (10^4^ Å^2^)	–	6.75	–		6.75	4.94	4.30	
〈*V*_α+β_〉 (10^7^ Å^3^)	–	17.06	–		17.06	10.96	9.03	
〈*V*_β_〉 (10^7^ Å^3^)	–	15.38	–		15.38	9.28	7.35	
φ_L_〈*V*_β_〉 ( 10^7^ Å^3^)	–	7.00	–		9.09	5.08	1.90	
*A*_Porod_ (10^3^ cm^−5^)	3.0	3.0	3.7		0.98	1.1	3.4	
*A*_DB_ (10^−20^ cm^−1^)	0.86	1.15	1.9		0.82	1.8	51	
*L*_DB_ (Å)	130	140	150		170	200	350	
*A*_OZ_ (10^−23^ cm^−1^)	6.5	9.0	30/5.0		6.0	6.5	46/9.0	
*L*_OZ_ (Å)	1.3	2.0	14/2.0		1.3	2.0	17/4.0	
*n* (10^−10^ Å^−3^)	17.6	13.9	5.40		17.9	16.5	6.25	
*n*^−1/3^ (Å)	828	896	1230		823	846	1170	

## Appendix A Decomposition of the partial scattering function

Contrast-variation experiments were conducted to obtain SANS profiles with different *P*_H,*i*_ (*i* = 1,…, *N*_prof_). From equation (17), the obtained profiles at various *P*_H_ constitute a system of linear equations, as described by the matrix

where the *N*_prof_ × 3 matrix in the second line is denoted by **M**. The matrix elements are contrast factors:

In our experiments, where the number of obtained profiles (*N*_prof_) is larger than 3, **M** is obtained as a nonsquare matrix (*N*_prof_ × 3). Therefore, instead of a simple inverse matrix, a Moore–Penrose pseudo-inverse matrix **M**^+^ is needed,

where **M**^T^ is the transposed matrix of **M**. **M**^+^ is known to give the shortest-length least-squares solution for equation (68):

## Appendix B Effect of the higher-order structure formed by aggregates

Considering the effect of the higher-order structure formed by aggregates, the spatial distribution functions  and  for regions α and α+β are calculated as

where **c**_*i*,agg_ is the aggregate center position, *i* is the labeling number of aggregates, and *N*_agg_ is the total number of aggregates in a sample. φ_α_(**r**) and φ_α+β_(**r**) are the spatial distributions of regions α and α+β considering the aggregates and the polymer adsorption layer and without considering a further higher-order structure. Hence, the form factors [ and ] and the cross term [] considering this higher-order structure are calculated as

Here, we define the structure factor 〈*K*(*q*)〉 as

The form factors considering this higher-order structure are calculated using 〈*K*(*q*)〉 as

Here, we assume that the aggregate spatial distribution is not affected by polydispersity in the aggregate structure. In this way, 〈*K*(*q*)〉 in equations (21)–(26) is derived.
