# Supplementary material for: Spin-contrast-variation small-angle neutron scattering study of fully and partially swollen silica-filled rubber
Source: J Appl Crystallogr. 2026 Mar 20;59(Pt 2):492–512. doi: 10.1107/S1600576726000361 (PMC13060475; doi:10.1107/S1600576726000361)
Supplement: Supplementary file 1 [file j-59-00492-sup1.pdf]

JOURNAL OF
APPLIED
CRYSTALLOGRAPHY

Volume 59 (2026)

Supporting information for article:

Spin-contrast-variation small-angle neutron scattering study of fully and partially swollen silica-filled rubber

Yohei Noda, Satoshi Koizumi, Tomomi Masui, Hiroyuki Kishimoto, Daisuke Yamaguchi, Takayuki Kumada, Shin-ichi Takata, Kazuki Ohishi and Jun-ichi Suzuki

Table S1 List of the symbols used in this study.

Symbol	Meaning	Equation
A_{DB}, A_{OZ}	Prefactor of the Debye-Bueche or Ornstein-Zernike function	30, 63
$A_{k,Np}$	Occurrence number of $B_{k,Np}$ in the double summation in Eq (46)	50
A_{Porod}	Prefactor of the q^{-4} term	63
b_H, b_D, b_C, b_N, b_O, b_S, b_{Si}	Scattering length of H, D, C, N, O, S or Si	1
$B_{k,Np}$	Possible distance between silica particle pairs for the N_p particle aggregate	50
$\mathbf{c}_{i,agg}$	Center position of silica aggregate	74-79
$\mathbf{c}_{i,Np}$	Center position of each silica particle in the N_p particle aggregate	42, 44
$C_{k,Np}$	Occurrence number of $D_{k,Np}$ in the summation in Eq (47)	51
$\mathbf{c}_{M,Np}$	Position of silica aggregate mass center position	43, 44
$D_{k,Np}$	Possible distance between silica particle and silica aggregate mass center for the N_p particle aggregate	51
$E_{Z,D}$, $E_{Z,H}$	Zeeman splitting energy of proton or deuteron	3
f_{silica}	Silica volume fraction	58-60
f_α, f_β, f_γ	Volume fraction of the regions α, β, and γ (Fig. 7)	56-60
$F_{sph}(q; R)$	Form amplitude of sphere with radius R	46-48, 49
$F_{\alpha,Np}(q)$, $F_{\alpha+\beta,Np}(q)$	Form amplitude for α or α+β regions for silica aggregate composed of N_p silica particles	46-48, 53-55
$\langle F_\alpha^2(q)\rangle$, $\langle F_{\alpha+\beta}^2(q)\rangle$	Form factor of the α or α+β region	21-26, 27, 28, 34, 35, 36, 53, 55, 61, 62, 64, 66, 67
$\langle F_\alpha(q)F_{\alpha+\beta}(q)\rangle$	Cross term between the α and α+β regions	22-26, 29, 34, 37, 54, 64, 66, 67
$\langle F_\alpha'^2(q)\rangle$, $\langle F_{\alpha+\beta}'^2(q)\rangle$	Form factor of the α or α+β region with considering higher-order structure formation by silica aggregates	76, 77, 80, 81
$\langle F_\alpha'(q)F_{\alpha+\beta}'(q)\rangle$	Cross term between the α and α+β regions with considering higher-order structure formation by silica aggregates	78, 82
$G_{Np}(R_p)$	Interference term for calculating $F_{\alpha,Np}^2(q)$	46, 50
$H_{Np}(R_p)$	Interference term for calculating $F_{\alpha,Np}(q)F_{\alpha+\beta,Np}(q)$	47, 51
$I(q; P_H)$	Polarization-dependent small-angle neutron scattering intensity	11, 17, 61, 68, 73

I_{inc}	Incoherent scattering intensity	6
<K(q)>	Structure factor for considering spatial distribution of aggregates	21-26, 61, 62, 66, 67, 80-82
$k_{max,G,Np}$	Maximum number of k in calculating $G_{Np}(R_p)$	50
$k_{max,H,Np}$	Maximum number of k in calculating $H_{Np}(R_p)$	51
L	Radius of spherical sum region composed of silica aggregate and surrounding polymer adsorption layer	39
L_{DB}, L_{OZ}	Correlation length of Debye-Bueche or Ornstein-Zernike function	30, 63
**M**	Contrast factor matrix, composed of $3 \times N_{prof}$ elements	68, 72
$\mathbf{M}^T$	Transposed matrix of **M**	72
$\mathbf{M}^+$	Moore-Penrose pseudo inverse matrix, composed of 3×3 elements	72, 73
n	Number density of aggregate	21-26, 61, 62, 66, 67
n_{PD}	Number of polymer-dense region per volume	-
N_{agg}	Total number of aggregates in a sample	74-79
N_p	Number of particles in one silica aggregate	40, 42-44, 46, 47, 50, 51
$N_{p,med}$	Median value of N_p	40
N_{prof}	Number of SANS profiles measured at different P_H	68, 73
N_{up}, N_{down}	Number of up- or down-spin protons	2
P_H, P_D	Spin polarization of proton or deuteron	1-4, 8-11, 17, 61, 68-71, 73
q	Magnitude of scattering vector	-[*1]
Q_{swell}	Ratio of the swollen rubber volume to its original one	58-60
**r**	Position vector	-[*2]
$r_{ij,Np}$	Distance between particle i and j centers in silica aggregate	46, 50
$r_{iM,Np}$	Distance between particle i center and silica aggregate mass center	47, 51, 52
R	Radius of sphere	45, 49
R_{agg}	Circumsphere radius of silica aggregate	39
$R_{agg,Np}$	Circumsphere radius of the model silica aggregate composed of N_p particle	43
$R_{g,\alpha}$, $R_{g,\alpha+\beta}$	Gyration radii of α or α+β regions	35-38
$R_{g,agg,Np}$	Gyration radius of the model silica aggregate composed of N_p particle	52
R_{N1}, R_{N2}, R_{N3}, R_{CA1}, R_{CA2}, R_{CA3}	Name of rubber samples	-

R_p	Radius of silica primary particle	41, 42, 46, 47, 50-55
$R_{p,med}$	Mean value of R_p	41
$S_{ij}(q)$	Partial scattering function between component i and j	12
$S_G(q)$	Scattering intensity due to the polymer chains in solvent	22, 23, 26, 30, 31-33, 61, 62, 66
$S_{G,Approx}(q)$	$S_G(q)$ contribution derived by use of Guinier approximation	65, 66
$S_{G,Porod}(q)$	Modified form of $S_G(q)$, in which DB function is replaced with q^{-4} term	63
$S_{PP}'(q)$, $S_{PP}'(q)$, $S_{PT}'(q)$	Self- or cross-correlation term of the partial scattering function for rubber samples without filler particles	31-33
$S_{SP}(q)$, $S_{ST}(q)$, $S_{PT}(q)$	Cross-correlation term of the partial scattering function	11, 14-17, 24-26, 34, 38, 62, 65, 68, 73
$S_{SS}(q)$, $S_{PP}(q)$, $S_{TT}(q)$	Self-correlation term of the partial scattering function for silica, polymer, or d-toluene component	11, 14-17, 21-23, 34, 38, 62, 65, 68, 73
t_L	Thickness of polymer adsorption layer	39, 43, 47, 48
t_s	Sample thickness	5, 6
T_N	Neutron transmission	5
V_{PD}	Volume of single polymer dense domain	-
V_s	Sample volume	27-29
$\langle V_\alpha \rangle$, $\langle V_\beta \rangle$, $\langle V_{\alpha+\beta} \rangle$	Expected volume of α, β or $\alpha+\beta$ regions	35-37, 38, 58-60
$W_{Np}(N_p)$, $W_{Rp}(R_p)$	Distribution function for N_p or R_p	40, 41, 53-55
$\Delta\varphi$	Polymer volume fraction difference between the polymer adsorption layer and the matrix	22-26, 34, 38, 56, 57, 62, 66, 67
$\Delta\varphi_{PD}$	Polymer volume fraction difference between polymer dense region and surrounding matrix	-
$\Delta_{SS}(P_H)$, $\Delta_{SP}(P_H)$, $\Delta_{PP}(P_H)$	Polarization-dependent contrast factors	68-71
2θ	Neutron scattering angle	-
λ	Neutron wavelength	-
ρ_{P+T}	Scattering length density of homogeneous polymer/d-toluene mixture	61
ρ_S, ρ_P, ρ_T	Scattering length density of silica, polymer or d-toluene	7-9, 10, 11,

		17, 61, 69-71
$\rho(\mathbf{r}; P_H)$	Position- and polarization-dependent scattering length density	10, 11
$\sigma_{inc,H}$, $\sigma_{inc,D}$	Microscopic incoherent scattering cross sections for H or D	-
σ_{Np}	Standard deviation of $\ln(N_p)$	40
σ_{Rp}	Standard deviation of R_p	41
$\sigma_{tot,H}$, $\sigma_{tot,D}$, $\sigma_{tot,C}$, $\sigma_{tot,N}$, $\sigma_{tot,O}$, $\sigma_{tot,S}$, $\sigma_{tot,Si}$	Microscopic total cross sections for H, D, C, N, O, S or Si	-
Σ_{inc}	Macroscopic incoherent scattering cross section	6
Σ_{tot}	Macroscopic total cross section	5
φ_{homo}	Polymer volume fraction outside the silica with assuming homogeneous mixture of polymer and d-toluene	56, 57
φ_L, φ_M	Polymer volume fraction at the polymer adsorption layer or the matrix	19, 20, 22-26, 34, 38, 56, 57, 62
$\varphi_S(\mathbf{r})$, $\varphi_P(\mathbf{r})$, $\varphi_T(\mathbf{r})$	Spatial distribution functions of the silica, polymer or d-toluene	10, 12, 13, 18-20
$\varphi_{sph}(\mathbf{r}; R)$	Spatial distribution of sphere with radius R	42, 43, 45
$\varphi_\alpha(\mathbf{r})$, $\varphi_\beta(\mathbf{r})$, $\varphi_\gamma(\mathbf{r})$	Spatial distribution functions of the regions α, β, and γ (Fig. 7)	18-20, 74
$\varphi_{\alpha+\beta}(\mathbf{r})$	Spatial distribution functions of the region $\alpha+\beta$ (Fig. 7)	28, 29, 75
$\varphi_{\alpha}'(\mathbf{r})$, $\varphi_{\alpha+\beta}'(\mathbf{r})$	Spatial distribution functions of α or $\alpha+\beta$ regions with considering higher-order structure formation by silica aggregates	74, 75
$\varphi_{\alpha,Np}(\mathbf{r})$, $\varphi_{\alpha+\beta,Np}(\mathbf{r})$	Spatial distribution functions of α or $\alpha+\beta$ regions for silica aggregate composed of N_p silica particles	42, 43

*1, 2 : Equation number is skipped for q and **r**, since q and **r** are contained in many equations.
